# Extracellular vesicles from type-2 macrophages increase the survival of chronic lymphocytic leukemia cells ex vivo

**DOI:** 10.1038/s41417-024-00802-7

**Published:** 2024-06-25

**Authors:** Léa Ikhlef, Nina Ratti, Stéphanie Durand, Rémy Formento, Héloïse Daverat, Marie Boutaud, Clément Guillou, Natalya Dmytruk, Nathalie Gachard, Pascal Cosette, Marie-Odile Jauberteau, Paul-François Gallet

**Affiliations:** 1 University of Limoges, UMR INSERM 1308, CAPTuR, Limoges, France; 2 PISSARO Proteomics Platform, Mont-Saint-Aignan Campus, Mont-Saint-Aignan, France; 3 Department of Clinical Hematology, University Hospital of Limoges, Limoges, France; 4 Hematology laboratory, UMR CNRS7276/ INSERM 1262, University Hospital of Limoges, Limoges, France; 5 Polymers, Biopolymers, Surface Laboratory, UMR 6270 CNRS, Normandie University, UNIROUEN, INSA Rouen, Mont-Saint-Aignan, France; 6 HeRacLeS-PISSARO, INSERM US 51, CNRS UAR 2026, Normandie University, Mont-Saint-Aignan, France; 7 Immunology laboratory, University Hospital of Limoges, Limoges, France

**Keywords:** Cancer microenvironment, Leukaemia

## Abstract

The resistance of Chronic Lymphocytic Leukemia (CLL) B-cells to cell death is mainly attributed to interactions within their microenvironment, where they interact with various types of cells. Within this microenvironment, CLL-B-cells produce and bind cytokines, growth factors, and extracellular vesicles (EVs). In the present study, EVs purified from nurse-like cells and M2-polarized THP1 cell (M2-THP1) cultures were added to CLL-B-cells cultures. EVs were rapidly internalized by B-cells, leading to a decrease in apoptosis (*P* = 0.0162 and 0.0469, respectively) and an increased proliferation (*P* = 0.0335 and 0.0109). Additionally, they induced an increase in the resistance of CLL-B-cells to Ibrutinib, the Bruton kinase inhibitor in vitro (*P* = 0.0344). A transcriptomic analysis showed an increase in the expression of anti-apoptotic gene *BCL-2* (*P* = 0.0286) but not *MCL-1* and an increase in the expression of proliferation-inducing gene *APRIL* (*P* = 0.0286) following treatment with EVs. Meanwhile, an analysis of apoptotic protein markers revealed increased amounts of IGFBP-2 (*P* = 0.0338), CD40 (*P* = 0.0338), p53 (*P* = 0.0219) and BCL-2 (*P* = 0.0338). Finally, exploration of EVs protein content by mass spectrometry revealed they carry various proteins involved in known oncogenic pathways and the RNAseq analysis of CLL-B-cells treated or not with NLCs EVs show various differentially expressed genes.

## Introduction

Chronic Lymphocytic Leukemia (CLL) is characterized by a progressive accumulation of monoclonal CD5^+^/CD19^+^/IgM^low^/IgD^low^ mature B-cells in the blood, bone marrow, lymph nodes and spleen, with an increased resistance to apoptosis and a low proliferation rate [1]. The CLL-B-cells proliferation rate is higher when the cells reside in lymphoid organs such as the bone marrow, lymph nodes, and spleen, but is lower in the peripheral blood [2]. This characteristic highlights that interactions between malignant B-cells and the tumor microenvironment (TME) play a major role in the physiopathology of CLL [3]. Within the TME, CLL-B-cells receive signals from various populations of accessory cells such as mesenchymal stromal cells (MSC), monocyte-derived nurse-like cells (NLCs), follicular dendritic cells (FDC), and T cells [4]. NLCs, which resemble tumor-associated macrophages, play a crucial role in CLL-B by creating a supportive microenvironment that sustains the homing, retention, and survival of CLL cells in secondary lymphoid organs [5]. They are able to promote CLL-B-cells survival notably through the secretion of APRIL, CXCL12, BDNF [6] and soluble CD14 or LFA-3/CD2 interaction [7]. Within the TME, cells communicate through the interaction of various surface and secreted proteins [8], but also through signals from extracellular vesicles (EVs) [9].

EVs are small vesicles known to mediate intercellular communications in local and distant microenvironments under physiological and pathological conditions. They are separated into two main categories; exosomes that are 30–150 nm vesicles generated through the formation of multivesicular bodies, and microvesicles that are 150–1000 nm vesicles resulting from the blebbing of the plasma membrane [10]. Crompot et al. highlighted the impact of bone marrow MSC EVs on CLL-B-cells in vitro [11] and showed that MSC-derived EVs are rapidly incorporated in CLL-B-cells and that they increase CLL cells migration, suggesting that these EVs could give CLL cells survival advantages in vivo. Recently, Gargiulo et al. also underscored the functional importance of EVs produced in the tumor microenvironment in promoting CLL progression by showing that TME EVs impair CD8^+^ T-cell mediated antitumor immune response and are indispensable for leukemia progression in vivo in murine models [12].

Although the impact of TME-derived EVs remains largely unknown, the impact of EVs from CLL-B-cells onto the TME is well documented. For instance, Ghosh evidenced that CLL-B-cells exosomes activate bone marrow stromal cells (BMSCs) *via* transfer of bioactive molecules including AXL receptor tyrosine kinase [13]. Research notably showed that EVs from CLL-B-cells induce an inflammatory phenotype in cancer-associated fibroblasts like cells [14] and increase immune synapse activity and migration of CD4^+^ T cells, possibly through the downregulation of T-cell immunoregulatory receptor CD69 [15]. Moreover, Farahani et al. showed that CLL-B-cells exosomes carry microRNA-202-3p that, once integrated in stromal cell lines, enhanced their proliferation and decreased apoptosis [16]. Overall, the literature suggests that CLL EVs target diverse cell types in the TME to reprogram their function and promote disease progression.

Here, we investigated the role of EVs derived from NLCs on different biological processes critical for CLL-B-cells survival. We found that EVs contribute to the survival of CLL-B-cells, notably by inducing the expression of anti-apoptotic proteins and enhancing their resistance to ibrutinib in vitro.

## Methods

### Primary culture

Isolation of peripheral blood mononuclear cells (PBMC) by density gradient was performed on blood samples from 16 patients (Supplementary Table 1) and 10 healthy donors. We used the MACSxpress Whole Blood B-CLL Cell Isolation Kit (#130104445, Miltenyi) to isolate B-cells from CLL patients. The viability and quantity of isolated B-CLL was assessed and they were then frozen in decomplemented exo-free serum supplemented with 10% DMSO [17]. After thawing, the cells were rested at 37 °C in RPMI 1640 medium (ThermoFisher) supplemented with 10% decomplemented fetal bovine serum (FBS) and 1% antibiotics (penicillin/streptomycin) for 1 h before starting any experiments.

### Cell line culture

The THP1 cell line (ATCC TIB-202) was cultured in RPMI 1640 medium (ThermoFisher) supplemented with 10% decomplemented FBS, 1% antibiotics (penicillin/streptomycin) and 0.1% β-mercaptoethanol (50 nM) (ThermoFisher). Cultures were performed at 37 °C, 5% CO_2_.

### Evaluation of cell proliferation and apoptosis

Apoptosis and proliferation were monitored using the IncucyteS3 (Sartorius), a live-cell imaging device enabling the tracking of fluorescence over set periods of time. It is notably used for evaluation of apoptosis and proliferation [18, 19]. Labeling of apoptotic cells was performed with Incucyte Annexin-V Dye for Apoptosis (Sartorius) and proliferation was evaluated either through nuclei labeling with NucLight (Sartorius) or using the Incucyte cell-by-cell tracking module to count cells. All images were acquired using the S3/SX1 G/R Optical Module, with the Non-Adherent Cell-by-Cell scanning mode, at x20, the acquisition time for green is of 300 ms and for red 400 ms, 4 images are taken per well. Analysis was performed with the Incucyte Base Analysis Software.

### Monocyte differentiation and polarization into M2 like macrophages

To differentiate THP1 into M2-like tumor-associated macrophages, 4.5.10^6^ cells were seeded in a T75 flask and treated with 50 ng/mL of Phorbol 12-myristate 13-acetate (PMA) for 24 h. Cells were then washed with PBS and the medium switched with fresh one, cells were incubated for 72 h. Medium was then replaced and supplemented with 20 ng/mL of IL-4 and IL-13 for 48 h [20, 21]. Cells were then rested in fresh medium for 24 h. Finally, the medium was switched with EVs-deprived medium, and cells were incubated for 48 h before supernatant collection. Efficient polarization into M2-macrophages was evaluated by confocal microscopy (Supplementary Fig. 1).

### Generation of CLL-blood-derived NLCs

NLCs were derived by culturing approximately 5.10^6^ cells of PBMCs from CLL patients in 2 mL of complete medium in 6-well plates. After 14 days, non-adherent CLL-B-cells were removed by vigorously washing each well with culture medium [22]. Efficient removal of CLL-B-cells was checked using flow cytometry (Supplementary Fig. 2). B-cells were removed each time to the best of our ability, but it is important to note that some residual B cells might subsist, however, they account for a negligeable amount, and our samples are significantly enriched in NLC, as shown on Supplementary Fig. 2. After the verification of NLC differentiation was conducted using confocal microscopy (Supplementary Fig. 1), the cells were incubated for 48 h in 2 mL of EVs-deprived complete medium. For subsequent experiments, culture supernatants from NLCs generated from different patients had to be pooled to obtain sufficient amounts of EVs.

### Isolation of monocytes from healthy donors

Blood from 10 healthy donors were centrifuged at room temperature for 25 min at 400xg. Plasma was discarded and buffy coats were collected in 15 mL tubes and diluted in PBS (1:1 dilution). Tubes were centrifuged for 10 min at 150 × *g* to pellet cells, which were then resuspended in 5 ml of PBS each. Then, 5 mL of Ficoll®-Paque Premium (Cytiva) was added to 15 mL tubes and the cell suspensions (5 mL) were gently layered over it. The tubes were centrifuged 30 min at 400 × *g* and the cells within a dense white mononuclear band were collected and diluted with 5 mL PBS. The cells were pelleted (300 × *g*, 5 min) and the supernatant was discarded. Cells were resuspended in 1 mL of exo-free medium and layered gently on 10 mL of Percoll® PLUS (Cytiva) in 15 mL tubes. The preparation was centrifuged at 580 × *g* for 15 min and monocytes within a band located at the interface of the medium and Percoll® solution were collected and diluted with 4 mL PBS. The cells were pelleted by centrifugation (550 × *g*, 10 min) and the supernatant was discarded. Cells were counted and viability assessed using Trypan blue staining. Monocytes were seeded onto 24-well culture plates at a density of 3.10^5^ cells/well in exo-free medium and transferred to an incubator (37 °C and 5% CO_2_) [23]. Monocytes were allowed to mature into a morphologically heterogeneous, adherent macrophage population over the course of 7 days. Afterwards, cells were analyzed by flow cytometry to check for the expression of monocyte/macrophage markers (Supplementary Fig. 3), and they were incubated for 48 h in 1 mL of EVs-deprived medium. Supernatants from healthy donors’ monocytes had to be pooled to obtain EVs in sufficient quantities for subsequent cell treatments.

### Flow cytometry analysis of immune cell markers

Cells were harvested, counted, and their viability was assessed using trypan blue staining before being seeded in a 96-well plate at a density of 100,000 cells per well. They were then washed using PBS 2% BSA and incubated with primary antibodies for 60 min at 4 °C. They were then washed again and fixed using 2% paraformaldehyde (ThermoFisher). They underwent a final wash and were resuspended in 200 μL of PBS 2% BSA. Fluorescence intensity was measured using the CytoFLEX LX device (Beckman Coulter) and data analyzed using the Kaluza software (Beckman Coulter). Antibodies used are listed in Supplementary Table 2. Raw flow cytometry data is available at the end of the supplementary data appendix.

### Isolation of EVs by ultracentrifugation

Culture supernatants were filtered through a 0.2 µm filter to remove contaminating apoptotic bodies, microvesicles, and cell debris before being centrifuged at 120,000 xg for 90 min to pellet EVs. Vesicles were resuspended with 50 mL PBS (ThermoFisher) [24] and centrifuged at 120,000 × *g* for 90 min using the Optima XPN-80 Ultracentrifuge (Beckman Coulter) with the 45 Ti Fixed-Angle Titanium Rotor (#339160, Beckman Coulter). The EV pellet was resuspended in of PBS for nanoparticle tracking analysis (NTA), in culture medium for cell treatments, or in RIPA buffer (ThermoFisher) for protein quantitation and western blot analysis. Protein concentration was determined using Bradford reagent (Biorad).

### Cell treatments with EVs

CLL-B-cells were seeded either in a 6-well plate at 2.10^6^ cells if they were destined for western blot analysis or in a 96-well plate at 25 000 cells for apoptosis and proliferation analyses. Cells were treated at a ratio of 1.10^-3 ^ng of EV protein per cell, resuspended in an ‘exo-free’ culture medium consisting of the previously described medium but supplemented with EVs-deprived FBS instead of standard FBS. To remove EVs, FBS was centrifuged at 120,000 × *g* for 16 h. The absence of EVs was verified by Nanoparticle tracking analysis (Supplementary Fig. 4).

### Nanoparticle tracking analysis (NTA)

NTA was performed using NanoSight NS300 (Malvern Panalytical Ltd.). Captures and analysis were achieved using the NanoSight Software NTA3.3.301. Samples were diluted in PBS and their concentration was adjusted by observing a particles/frame rate of around 50 (30–100 particles/frame). For each measurement, five consecutive 60 s videos were recorded (cell temperature: 25 °C, syringe speed: 22 μl/s).

### Labeling and internalization of EVs

To check for the ability of EVs to penetrate cells, EVs were labeled with PKH67 according to the PKH67 Green Fluorescent Cell Linker Kit (#PKH67GL, Merck). Freshly isolated EVs were resuspended in 1 mL of diluent C provided in the kit. As a control, in another ultracentrifuge tube, 500 μL of exo-free medium was brought up to 1 mL using diluent C as well. Then, 6 μL of PKH67 dye was added into each tube and mixed continuously for 30 s by gentle pipetting. After 5 min of incubation at room temperature, 2 mL of 10% BSA in PBS solution was added to quench the reaction and the volume was brought up to 8.5 mL with serum-free medium. After addition of 1.5 mL of 0.971 M sucrose, the tubes were centrifuged at 190,000 xg for 2 h at 4 °C. The media and interface layer were removed, and the EVs pellet was resuspended in PBS through gentle pipetting. CLL-B-cells were then exposed to either labeled EVs or exosome-free medium which underwent identical treatment steps as the EVs sample. The internalization of these components was assessed using the 488 nm excitation laser of the CytoFLEX flow cytometer (Beckman Coulter), and the results were analyzed using the Kaluza software (Beckman Coulter). Raw flaw cytometry data is available in the supplementary data appendix.

### Confocal microscopy

The internalization of labeled EVs into CLL-B-cells was assessed using both flow cytometry and confocal microscopy. For confocal microscopy analysis, 100 000 cells were seeded onto 8-well glass slides (Millicell EZ SLIDE, Merck Millipore) previously coated with a 0.01% poly-L-ornithine solution (Sigma Aldrich). The cells were then either treated with PKH67-labeled EVs or exposed to exo-free medium which underwent the same PKH67 labeling protocol for 2 h. After treatment, the cells were washed with ice-cold PBS and fixed in 2% paraformaldehyde (ThermoFisher) for 15 min at room temperature. Subsequently, they were washed again with PBS and the slide was mounted with a drop of mounting medium containing DAPI (Sigma) and a coverslip. The slides were allowed to rest for 15 min in the dark before proceeding to confocal microscopy analysis. Imaging was performed using an LSM880 confocal microscope (Zeiss) equipped with a 488 nm laser at a magnification of x63, and the acquired data were analyzed using Zeiss ZEN software. Confocal microscopy was also used to check for the expression of M2-subtype macrophages markers on M2-polarized THP1 cells and patient-derived NLCs. To do so, cells cultured on coverslips within 12-well plates were fixed using 2% paraformaldehyde (ThermoFisher) for 15 min at room temperature. After incubation, cells were blocked with PBS 1% BSA for 1 h at room temperature and then incubated with fluorochrome-coupled primary antibodies (anti-CD68 Alexa Fluor 647 #51-0689-42, Invitrogen & anti-CD163 Alexa Fluor 488 #568188, BD Biosciences) at a dilution of 1/100 for 1 h at 4 °C in the dark. Cells were then washed and mounted on slides using DAPI containing mounting medium. Slides were observed using LSM880 confocal microscope (Zeiss) at x40 magnification and data analyzed using Zeiss ZEN software and ImageJ.

### Western blot assay

For Western blot analyses, cells and/or extracellular vesicles (EVs) were lysed using RIPA buffer (Sigma-Aldrich) supplemented with 1% protease inhibitor cocktail (Cell Signaling). The lysates were sonicated using a Vibra-Cell Sonifier and then centrifuged for 20 min at 16,000 × *g* and 4 °C. The resulting supernatants were collected, and the protein concentration was quantified using Bradford reagent (Biorad). Samples were prepared in 1X Laemmli buffer (Biorad) supplemented with 10% beta-mercaptoethanol and denatured for 5 min at 95 °C. The proteins were then separated on a 12% SDS-PAGE gel, transferred to a polyvinylidene difluoride (PVDF) membrane, and blocked for 1 h in a PBS-BSA 5% (w/v) solution. Subsequently, the membrane was probed overnight at 4 °C with specific primary antibodies (refer to Supplementary Table 2). After overnight incubation, the membranes were washed and then incubated with the appropriate secondary antibody for 1 h. Membranes were visualized using Immobilon ECL Ultra (Merck Millipore, MA, USA) and the Azure 400 imaging system (Azure Biosystems). The acquired images were analyzed using ImageJ to conduct densitometric analyses. The relative densitometry was determined by dividing the densitometry of the band corresponding to the protein of interest by the densitometry of the band representing the housekeeping protein used as an internal control.

### Antibody array

CLL-B-cells, either treated with NLCs EVs or left untreated, were lysed using RIPA buffer supplemented with 1% protease inhibitor cocktail. The lysates were sonicated and centrifuged for 20 min at 16,000 × *g* and 4 °C. Following quantification of protein concentration using Bradford reagent, 200 µg of cell lysate proteins from cells treated or untreated with NLCs EVs were used to analyze the expression of the main apoptosis actors. This analysis was performed using the Human Apoptosis Antibody Array (#ab134001, Abcam).

### RNA extraction and RTqPCR analysis

Total RNAs were extracted from cells using the Quick-RNA™ Microprep Kit (Zymo Research) and quantified by NanoDrop 2000 (Thermo Fischer). For RT-qPCR analyses, 2 µg of RNA were reverse-transcribed into cDNA using the cDNA Archive kit (Applied Biosystems). Quantitative gene expression was performed using SensiFAST Probe Hi-ROX kit (Bioline, London) on a QuantStudio 5 (Thermo Fischer). Results were normalized to actin and 18 S expression and analyzed using the ∆∆^Ct^ method.

### Transcriptomic profiling by RNASeq

mRNA from B-cells from five CLL patients that were either treated with NLCs EVs or left untreated (paired control samples), were sequenced by Novogene Company (UK) according to the manufacturer’s protocol. Quality control of 150 bp paired-end reads were checked by FastQC software, low-quality end reads were trimmed (Phred quality score <30), and adapter removal was performed using Cutadapt [25]. The clean reads of each sample were mapped to the human reference genome (GRCh38 assembly) using STAR [26] splice-mapper and read counts for each gene were obtained using the featureCounts [27] counting tool. Mapping quality analysis revealed that two matched patients’ samples (EVs-treated and control) failed to obtain a good mapping rate (<70%) and were discarded for subsequent analysis [28].

Differential gene expression was conducted pair-wise using DESeq2 [29] to identify differentially expressed genes (DEGs) in EVs-treated versus untreated NLCs according to the following selection criteria: absolute fold change superior to 1,5 and *p*-adjusted (after Benjamini-Hochberg (BH) multiple testing correction) inferior to 0.1.

### Sample preparation for LC MS/MS and whole proteome analysis

Sample preparation and whole proteome analysis of EVs was performed by the PISSARO proteomics platform (Rouen, France). EVs proteins (*n* = 3) were resuspended in a dedicated solubilization buffer and protein concentration was estimated by Bradford assay (Biorad). An “in gel” tryptic digestion on 10 µg of the total protein extract was performed to obtain the subsequent peptide fraction used for mass spectrometry experiments. Peptides fractions were solubilized in Formic Acid 0.1% and analyzed on a Q-Exactive Plus Orbitrap^TM^ mass spectrometer (Thermo Scientific) coupled to a chromatographic system, Easy nanoLCII system (Thermo Scientific) All spectra obtained were exported in “raw” format to identify peptides and proteins with Proteome Discoverer 1.4 Software (Thermo Scientific). Peak lists were searched using MASCOT search software (Matrix Science) against the *human* Swissprot database.

To analyze the data, we started by using Venny [30] to generate a Venn diagram using our three replicates, we kept the proteins found in common, which represented 56.6% of the total amount. We then used DAVID [31] to perform functional enrichment according to REACTOME terms and only retained entries with *p* values inferior to 0.05 using the Benjamini-Hochberg correction to determine the false discovery rate.

### Statistical analyses

Statistical analyses were performed using GraphPad Prism 9.0. For Incucyte analyses, the area under curve (AUC) of each condition was determined and the values obtained compared using non-parametric multiple comparisons tests (Friedman test). For RTqPCR and western blot analyses, values were also compared using non-parametric tests. *P* values ****P < 0.0001, ***P < 0.001, **P < 0.01, *P < 0.05 were considered significant. “ns” signifies “not significant”, to make some of the graphs more legible, only significant *p* values appear, when there is no asterisk, it means that the *p* value is higher than 0.05.

## Results

### Patient-derived NLCs and M2-THP1 cells produce EVs which are rapidly incorporated into CLL-B-cells

As the percentage of apoptotic cells reaches around 68% after 8 days of culture, it decreases to around 16% when CLL-B-cells are co-cultured in the presence of nurse-like cells (NLC), and to approximately 28% when CLL-B-cells are co-cultured in the presence of M2-THP1 cells (Supplementary Fig. 5). As previously demonstrated, these data show that both NLCs and M2-THP1 cells facilitate the survival of leukemic B-cells [4, 32]. In the present study, we hypothesize and provide evidence that both NLCs and M2-THP1 are able to send survival signals to leukemic cells through EVs.

**Fig. 1:**
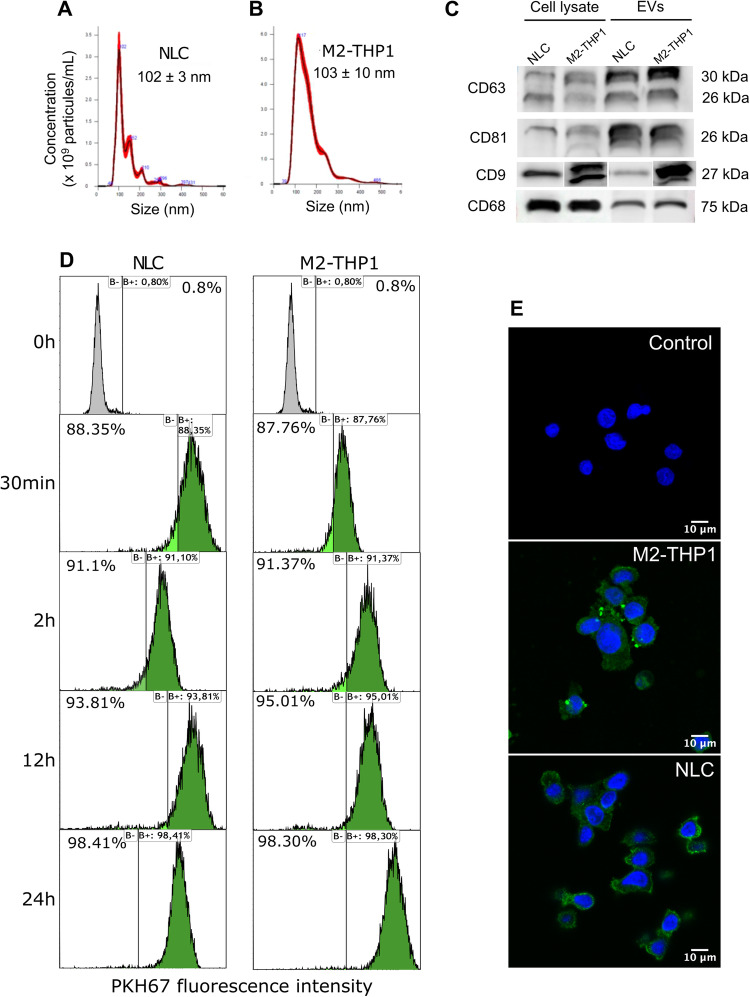
NLCs and M2-THP1 cells release extracellular vesicles into their growth medium, which are efficiently incorporated into CLL-B-cells. **A** EVs were isolated by ultracentrifugation from Nurse-Like Cells (NLCs) culture medium, their size was evaluated (mode (nm) ±SEM) by NTA using the NanoSight NS3000 (Malvern Panalytical). In (**B**), a similar analysis was conducted for EVs isolated from M2-THP1 culture medium. **C** Western blot analysis was performed to assess exosomal (CD9, CD81, and CD63) and macrophage (CD68) markers on NLCs and M2-THP1 cells lysate and EVs isolated from the culture medium of NLCs and M2-THP1 cells. **D** Time-course analysis by flow cytometry of PKH67 labeled EVs incorporation into CLL-B-cells (0.001 ng of EVs per cell). **E** Fluorescence microscopy of CLL-B-cells incubated for 2 h with PKH67-labeled EVs isolated from the culture medium of NLCs and M2-THP1 cells, or without EVs (x63 magnification).

To investigate, we first verified the presence of EVs in the culture supernatants of NLCs and M2-THP1 cells. EVs were isolated by ultracentrifugation and their size distribution was analyzed by nanoparticle tracking analysis (NTA). A mode of 103 ± 10 nm was obtained for the size of both types of EVs (Fig. 1A, B), signifying that the isolated vesicles are primarily in the size range of exosomes [33]. EVs were also analyzed by immunoblots directed against CD9, CD63 and CD81. These three exosomal markers [34] were present in the protein extracts from vesicles, further confirming the possibility that these EVs are enriched in exosomes (Fig. 1C). EVs also expressed macrophage marker CD68, thereby confirming their source (Fig. 1C).

Next, to check for the ability of the EVs to penetrate CLL-B-cells, they were incubated for 30 min, 2 h, 12 h or 24 h with PKH67 green-labeled EVs obtained from NLCs or M2-THP1 cells, and the green fluorescence was analyzed by flow cytometry (Fig. 1D). The uptake of EVs was a rapid process with more than 87% of CLL-B-cells having integrated fluorescent EVs after 30 min. The integration of EVs was also confirmed by confocal microscopy imaging of CLL-B-cells after 24 h of incubation (Fig. 1E).

### EVs from NLCs and M2-THP1 protect CLL-B-cells from spontaneous apoptosis ex vivo

The addition of EVs from NLCs and M2-THP1 cells to CLL-B-cells resulted in a decrease of spontaneous apoptosis of CLL-B-cells. The rate of CLL-B-cells undergoing apoptosis over a period of 48 h decreased significantly with the addition of EVs (Supplementary Fig. 6). Surprisingly, the decrease of apoptosis level was equivalent for all the EVs quantities tested (10–100 ng of EVs proteins).

The addition of EVs also affected the proliferation of CLL-B-cells ex vivo. As shown on Supplementary Fig. 6, the proliferation rate (calculated over a period of 48 h) significantly increased following the addition of EVs, reaching a plateau at 25 ng. This data underlines the strong anti-apoptotic effect of EVs, even at relatively low dosage, as well as their proliferative effect. For the rest of the experiments, we chose to treat CLL-B-cells with 25 ng of EVs protein per 25 000 cells (corresponding to a concentration of 1.10^-3^ ng/cell), as it was the lowest concentration we tested that had the highest significant effect for both apoptosis and proliferation.

**Fig. 2:**
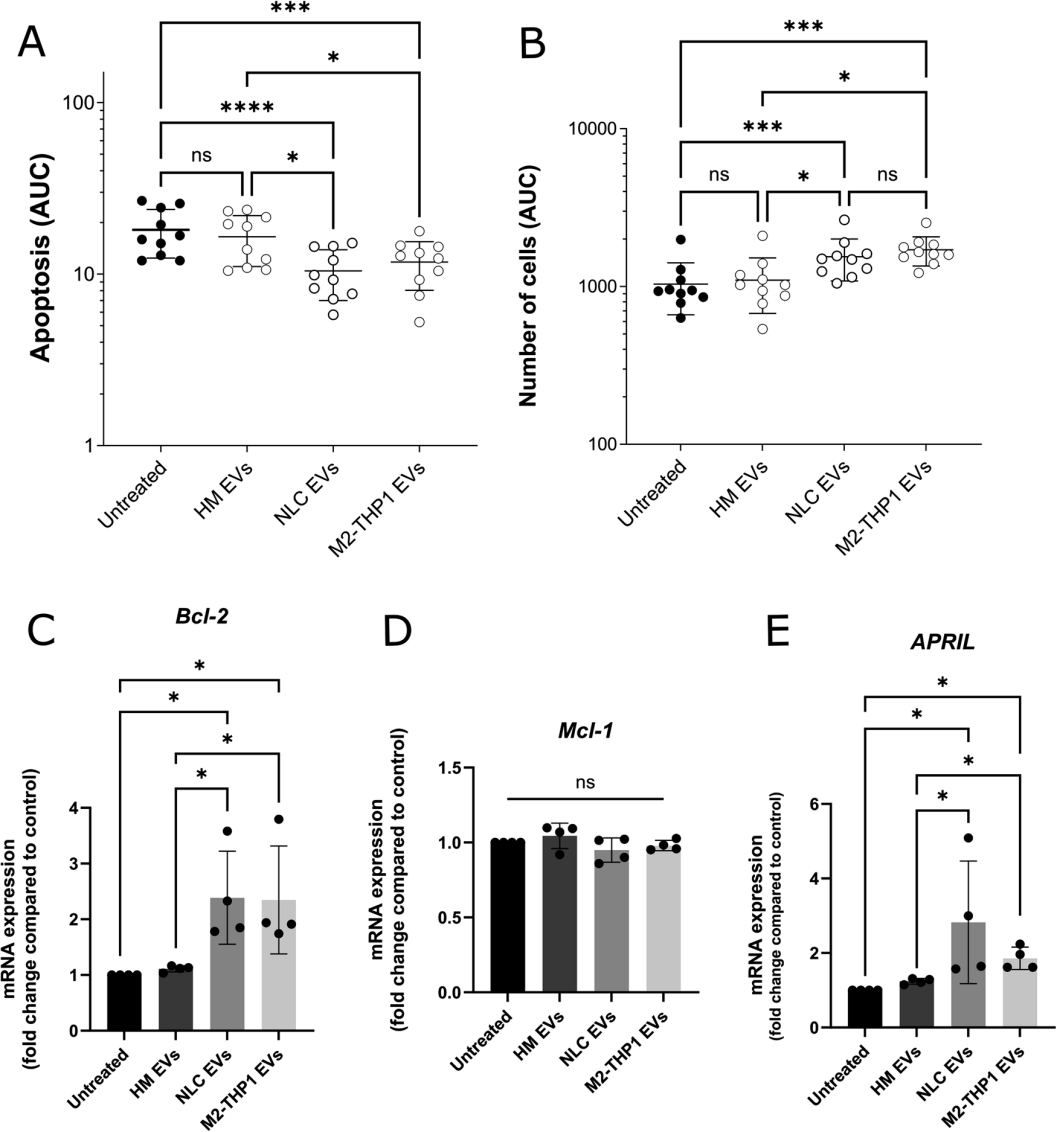
EVs from both M2-THP1 and NLCs protect CLL-B-cells from apoptosis and promote their proliferation ex vivo. Experiments were conducted using the IncucyteS3 live-cell imaging device, 25 000 CLL-B-cells were treated with 25 ng of EVs from either NLCs or M2-THP1 cells and marked with Annexin V (Sartorius) and fluorescence was monitored for 48 h. **A** The percentage of cells in apoptosis and (**B**) the proliferation rate were determined using the Incucyte analysis software. The area under curve (AUC) of each condition was calculated and they were compared with a Friedman non-parametric test. Experiments were repeated on CLL-B-cells from 10 different patients (patients #1-10 - Supplementary Table 1), each time in technical triplicates. Results are presented as mean ± SEM. **C**–**E** BCL-2, MCL-1 and APRIL relative expression in CLL-B-cells incubated without or with EVs isolated from healthy monocytes/macrophages (HM); NLCs and M2-THP1 cells culture medium. The RTqPCR were performed on three different individuals (patients #1, 3 and 4 - Supplementary Table 1), each time in technical replicates using 18 S as endogenous control. Results were compared with a Kruskal-Wallis test and presented as mean ± SEM.

The analyses conducted using the IncucyteS3 device enabled us to monitor apoptosis and proliferation levels over a 48-hour period. As a control, EVs from healthy donors’ monocytes/macrophages (NLCs are only associated to CLL) were isolated (Supplementary Fig. 3) to assess the impact of healthy counterparts of NLCs and eliminate the possibility that the observed effects could result from EV internalization, regardless of the source. As expected, there was no significant effect of EVs isolated from healthy monocytes/macrophages on CLL-B-cell apoptosis and proliferation. However, the addition of EVs from M2-THP1 and NLCs (25 ng proteins per 25,000 cells) resulted in a significant decrease in apoptosis (Fig. 2A), while CLL-B-cell proliferation increased under the same conditions (Fig. 2B)

As resistance to apoptosis is a hallmark of CLL-B clonal lymphocytes, characterized by elevated expression of anti-apoptotic BCL-2 family proteins [34], we measured the expression levels of *BCL-2* and *MCL-1* genes, which encode anti-apoptotic proteins, in CLL-B-cells incubated with EVs from M2-THP1 cells or NLCs (Fig. 2C, D). *MCL-1* gene expression remained unaffected by EVs (Fig. 2D), while BCL-2 was significantly overexpressed in CLL-B-cells incubated with EVs (Fig. 2C). Additionally, *APRIL* (a proliferation-inducing ligand) has been previously described as overexpressed in CLL-B-cells [35]. Therefore, we evaluated the expression level of this growth factor in CLL-B-cells incubated with EVs from M2-THP1 cells or NLCs (Fig. 2E) and observed that EVs induced an overexpression of *APRIL* in CLL-B-cells.

### EVs from NLCs activate anti-apoptotic pathways in CLL-B-cells

**Fig. 3:**
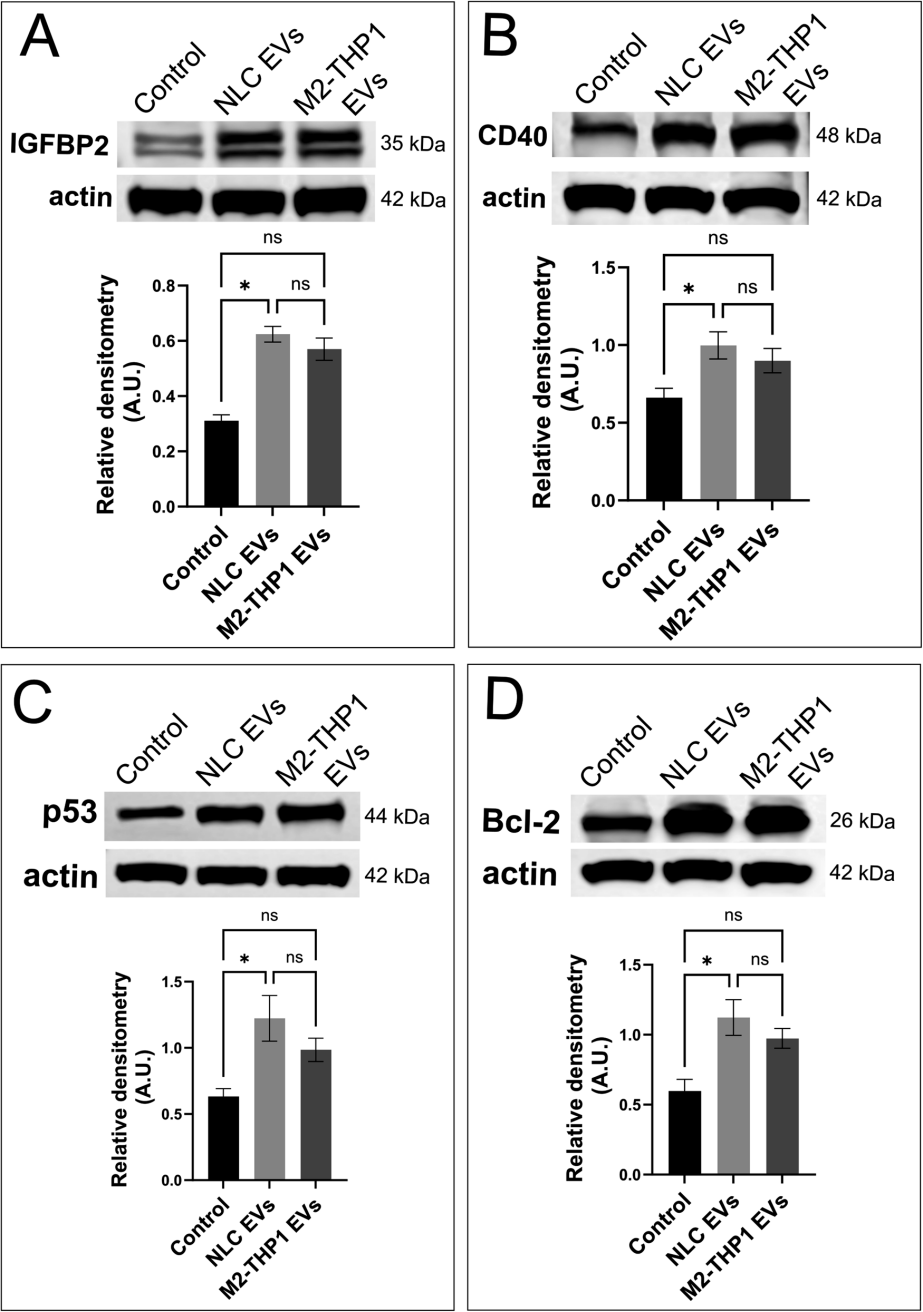
EVs from NLCs and M2-THP1 increase the expression of apoptosis-related proteins. CLL-B-cells from 3 different patients (patients #4, 6 and 11 - supplementary table 1) were treated with EVs derived either from NLCs or M2-THP1 cells, after 24 h, cells were harvested and cell lysates were subjected to western blot analysis. **A** Immunoblot anti-IGFBP2, (**B**) Immunoblot anti-CD40 (**C**) Immunoblot anti-p53 and (**D**) an immunoblot anti-BCL2. Densitometric analyses are represented under blots. In all cases, the actin signal was used for normalization. Results were compared with a Kruskal-Wallis test and are presented as mean ± SD. Immunoblots presented are representative of three independent experiments.

We previously observed that when incubated with EVs derived from NLCs and M2-THP1 cells, CLL-B-cells demonstrated enhanced survival, a process already described as dependent on the overexpression of BCL-2 [6]. To gain a deeper understanding of the anti-apoptotic pathways triggered by those EVs, we conducted a comprehensive analysis of apoptotic pathways activation using a protein array approach (Supplementary Fig. 7). The array we employed allowed us to quantify the relative levels of 43 proteins associated with apoptosis. Comparative analysis between untreated CLL-B-cells and those incubated with EVs from patient-derived NLCs revealed a significant increase in the levels of IGFBP-2 and CD40 proteins following EVs treatment. There was also an increase in the amount of BCL-2 and p53 proteins, whose involvement in CLL has already been well documented [35, 36], but it was not significant. In order to better confirm these variations in protein levels, we analyzed CLL-B-cells from three different patients treated or not with NLCs EVs (Fig. 3). The results confirm a significant increase in the levels of proteins IGFBP-2, CD40, p53, as well as BCL-2 in CLL-B-cells incubated with NLC EVs.

### EVs from NLCs affect gene expression in CLL patients

**Fig. 4:**
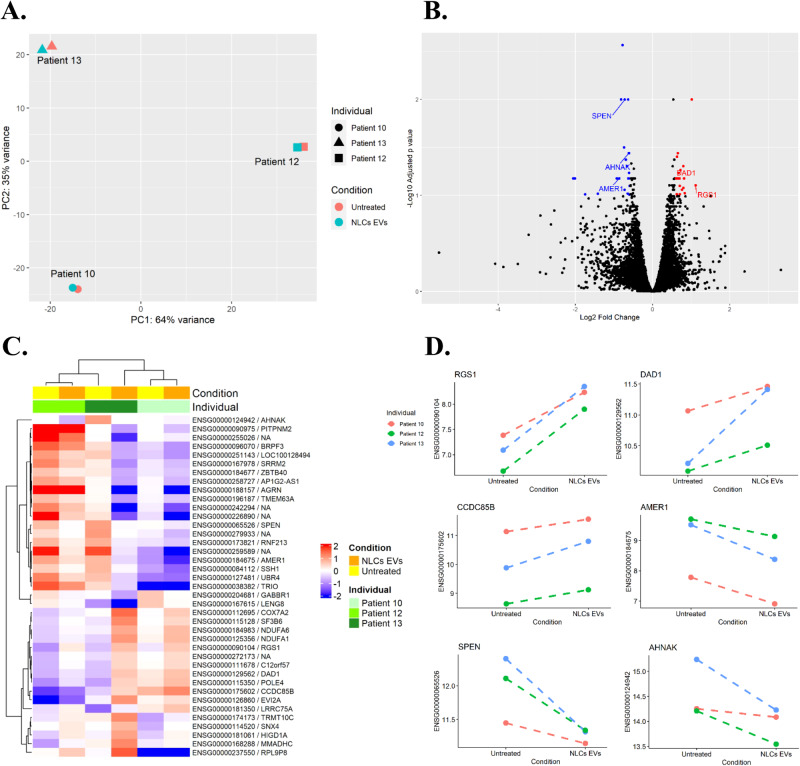
NLCs EVs induce gene expression changes in CLL-B-cells. **A** Principal component analysis (PCA) of RNAseq data transcripts of patients’ CLL-B-cells treated (“NLCs EVs”) or not (“Untreated”) with NLCs EVs. (Patients #10, 12 & 13- Supplementary Table 1). **B** Volcano plot showing differential expression of genes (DEG) with FDR < 0.05 in CLL-B-cells from patients treated with NLCs EVs. **C** Heatmap displaying the results of a hierarchical clustering of all differentially expressed genes between CLL-B-cells treated with NLCs EVs versus those untreated. **D** Differential expression of selected genes following NLCs EVs treatment of CLL-B-cells.

The elevated levels of anti-apoptotic proteins observed in CLL-B-cells when incubated with NLCs EVs prompted us to explore the transcriptome alterations in the treated B-cells in comparison to untreated cells. To achieve this, we performed an RNAseq analysis on B-cells obtained from five distinct CLL patients that were either treated with NLCs EVs or left untreated. Unfortunately, the analysis was inconclusive for two out of the five patients due to issues during sequencing (significantly less reads than the other samples with over 30% of reads mapped only once), so we chose to analyze the data for only three patients (patients #10, 12 and 13). Due to the small size of the cohort and high inter-individual variability (Fig. 4A), we analyzed results using a paired-analysis method. A list of the 39 differentially expressed genes, 17 upregulated and 22 downregulated, between the treated *versus* the control conditions, is available in Supplementary Table 3. Interestingly, 77% of DEGs were protein-coding genes and 15% were lncRNA.

Among the upregulated genes, we notably found: *RGS1* (Regulator of G protein Signaling 1) and *DAD1* (Defender against cell death 1), as shown on Fig. 4D.

RNASeq results also revealed various downregulated genes (Fig. 4B). Among them, we found *SPEN* (spen family transcriptional repressor); *AHNAK* (AHNAK nucleoprotein) and *AMER1* (APC membrane recruitment protein 1).(Fig. 4D).

Overall, these results suggest that EVs from patient-derived NLCs are able to induce gene expression modifications in CLL-B-cells that could promote CLL progression through the activation or inhibition of diverse signaling pathways involved in oncogenesis. However, it is important to note that due to the small number of individuals included in the study and high inter-individual variability, these results remain preliminary. Although they provide valuable insight into the potential of NLCs EVs to alter gene expression in order to transfer aggressiveness to CLL-B-cells, there is a need for further experimental work both to confirm and deepen our understanding of the observed changes.

### M2-THP1 EVs contain proteins involved in various oncogenic pathways

In order to identify which protein actors could be responsible for transferring aggressiveness to CLL-B-cells, we conducted a whole proteome analysis on EVs using mass spectrometry. We were unable, due to technical difficulties (to obtain sufficient quantities of EVs protein extract from our NLCs cultures), to perform this analysis on EVs derived from NLCs, so we used EVs from M2-THP1 cells, as they were easier to obtain in quantities sufficient for subsequent mass spectrometry analysis.

**Fig. 5:**
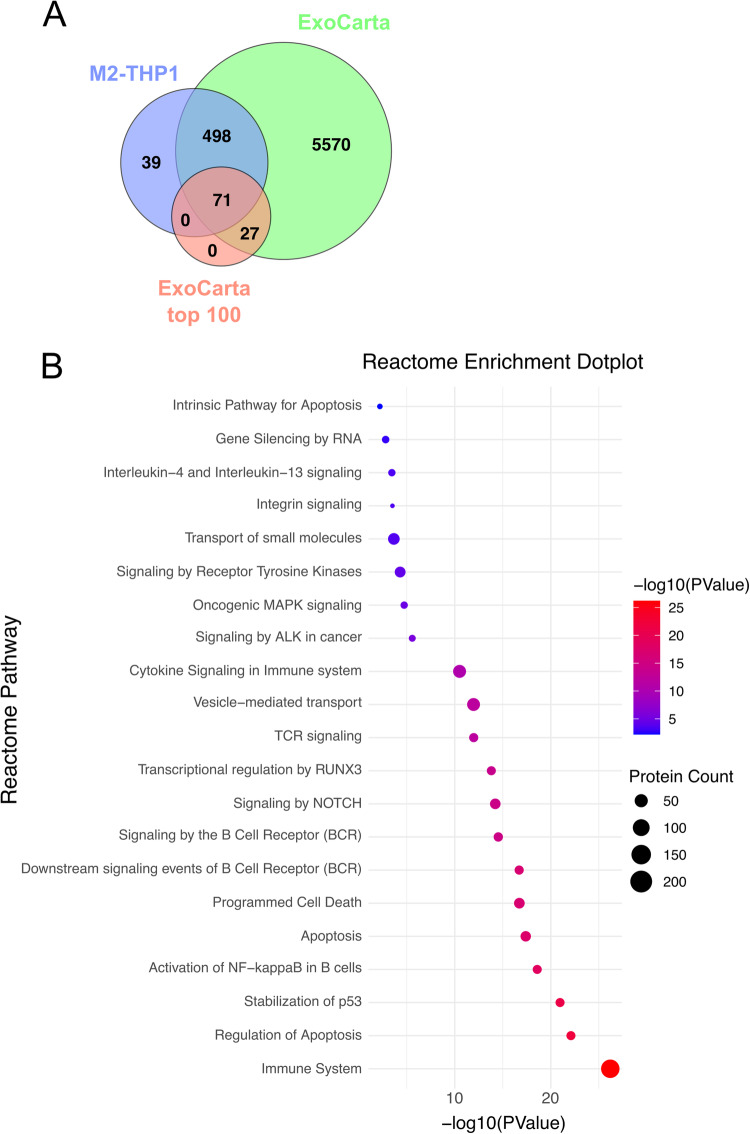
M2-THP1 EVs contain proteins implicated into apoptosis resistance. EVs isolated from M2-THP1 culture medium were isolated and MS² analyzed to identify their protein content. **A** Venn diagram of the identified proteins against the ExoCarta database. **B** Reactome enrichment dotplot of some of the relevant pathways identified. The data presented refers to proteins identified into three independent EVs preparations. The complete list of proteins identified is presented in Supplementary Table 4.

Proteomics analysis identified more than 1000 proteins in the 3 replicates, from which 608 proteins were common to all samples (Supplementary Table 4). The whole proteome analysis of M2-THP1 EVs identified several pathways that have been linked to hematological malignancies, and in some cases, specifically to chronic lymphocytic leukemia. In Fig. 5A, we generated a Venn diagram of the proteins that were identified in each replicate by mass spectrometry in M2-THP1 EVs against the human protein repertoire of the database ExoCarta [37]. This shows that 93.6% of the proteins we identified are within the ExoCarta database and that 71% of the most-found proteins within exosomes (ExoCarta top 100) are present within the samples, further confirming that there are in fact exosomes within the samples. To gain insight into the function of the identified proteins, a REACTOME enrichment dot plot was created (Fig. 5B). It facilitated the identification of several pathways of interest within the context of our study, all of which exhibited significant *p* values.

The list of all identified pathways and associated genes as well as the list of all the proteins identified by mass spectrometry are available in the supplementary data appendix (Supplementary Table 4). Among the pathways identified, we unsurprisingly found pathways relating to the immune system, T cell receptor (TCR) signaling, interleukin signaling and BCR signaling. Dysregulation of the immune system is a key factor in the development of hematological malignancies, as it can lead to the loss of tolerance to self-antigens and the accumulation of malignant cells [38]. These results are in accordance with previous work which evidenced that proteins involved in those same pathways are overexpressed in EVs from the TME of murine CLL models compared with healthy controls [12]. Meanwhile, BCR signaling plays a crucial role in the survival and proliferation of B-cells, dysregulation of its signaling has been implicated in the pathogenesis of CLL and many other B cell malignancies.

In accordance with results obtained within this study, many proteins involved in the regulation of apoptosis were identified. Additionally, we found actors involved in the activation of transcription factors such as NF-κB and RUNX3, which have both been implicated in the development of hematological malignancies [39, 40].

Furthermore, many proteins involved in the stabilization of p53 were found. The p53 pathway is a critical tumor suppressor pathway that prevents the accumulation of unrepaired DNA damage. In CLL, p53 alterations are associated with poor clinical outcomes and treatment failures [41, 42].

In summary, the pathways identified in the proteomic analysis of EVs produced by M2-THP1 cells are involved in various aspects of hematological malignancies, including immune regulation, cell survival, and cell death. These pathways have been linked to the development and progression of CLL and other hematological malignancies, highlighting the potential importance of EVs in the pathogenesis of these diseases.

### EVs from NLCs cells increase resistance to Ibrutinib of CLL-B-cells

**Fig. 6:**
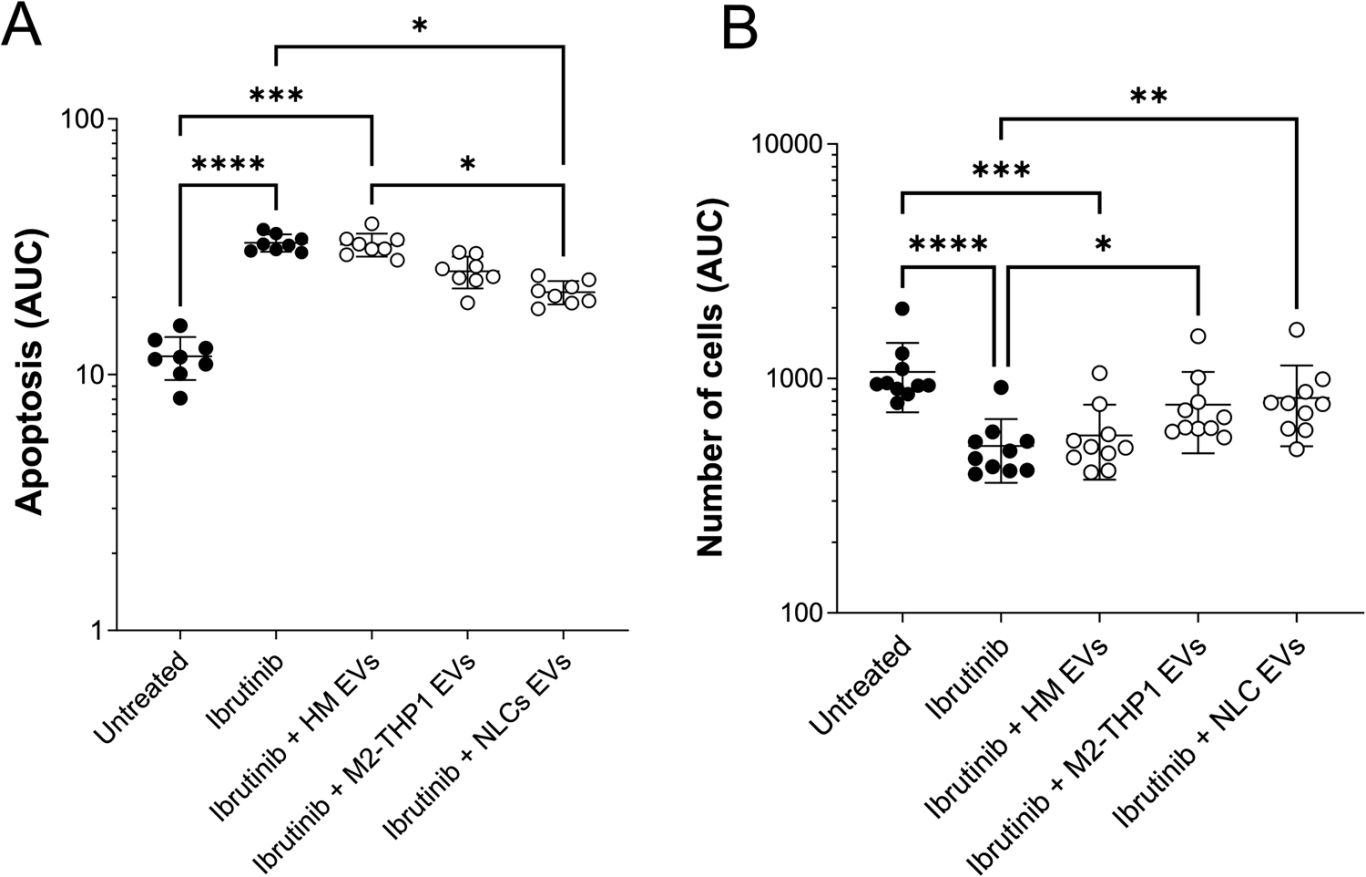
EVs from NLCs and M2-THP1 cells culture increase resistance to Ibrutinib of CLL-B-cells ex vivo. 25 000 CLL-B-cells from 8 different patients (patients #5, 8, 9, 11-16 - Supplementary Table 1) were incubated without or with EVs isolated from healthy monocytes/macrophages (HM); NLCs and M2-THP1 cells culture medium (25 ng of EVs protein for 25 000 cells) before 0.1 µM Ibrutinib was added. **A** The percentage of cells in apoptosis and (**B**) the proliferation rate were determined using the Incucyte analysis software. The areas under curve (AUC) of each condition were calculated and were compared with a Friedman non-parametric test. Experiments were repeated each time in technical triplicates. Results are presented as mean ± SEM. Only significant *p* values appear, when there is no asterisk, it means that the *p* value is higher than 0.05.

As seen before, EVs induce resistance to apoptosis, but we wondered whether they were implicated in CLL-B-cells drug resistance, notably resistance to Ibrutinib. CLL-B-cells from eight different patients were treated with either Ibrutinib alone, Ibrutinib in combination with EVs from healthy monocytes/macrophages, Ibrutinib in combination with EVs from patient-derived NLCs or Ibrutinib in combination with EVs from M2-THP1 cells. Following treatments, there was no observable effect of healthy monocytes/macrophages. CLL-B-cells treated with both Ibrutinib and NLCs EVs showed a significantly lower (*P* = 0.0344) apoptosis level compared to those treated with their healthy counterparts, with a 1.4-fold decrease in apoptosis level (Fig. 6A). As for M2-THP2 EVs, although not significant, there was a slight decrease in apoptosis as well compared to the condition treated with Ibrutinib and healthy monocytes-derived EVs.

Regarding proliferation, whereas not statistically significant, results tended to show an increase in cells treated with both Ibrutinib and NLCs EVs as well as cells treated with both Ibrutinib and M2-THP1 EVs compared to cells treated with Ibrutinib and healthy monocytes/macrophages.

As a whole, these results suggest that EVs derived from NLCs are able to induce Ibrutinib resistance in patients leukemic B-cells in vitro.

## Discussion

Extracellular vesicles are membrane-encapsulated particles that mediate cell-to-cell communication by transferring regulatory molecules such as proteins, lipids, and nucleic acids between cells [43]. In the context of hematological malignancies, EVs have been shown to contribute to the development and progression of the disease by promoting cancer cell survival, growth, and invasion [44]. They have been shown to carry oncogenic molecules, modulate the immune system, promote angiogenesis, and induce stroma remodeling [45]. Furthermore, EVs have potential applications in cancer diagnosis and therapy, as their molecular cargo reflects the nature and status of the cells of origin, making them interesting tools to identify new specific therapeutic targets, improve prognosis assessment [46] or even be used as therapeutic carriers [47]. EVs therefore play a crucial role in hematological malignancies by mediating intercellular communication and influencing various aspects of cancer development and progression.

In the present study, we investigated the role of EVs as communication tools between the microenvironment, specifically nurse-like cells, and leukemic B-cells. We showed that EVs derived from NLCs, as well as their in vitro model equivalent, M2-polarized THP1 cells, protect CLL-B-cells from apoptosis and enhance their proliferation ex vivo compared to EVs derived from healthy monocytes, notably through the overexpression of anti-apoptotic gene *BCL-2* and proliferation-inducing gene *APRIL*.

We also showed that following EVs treatment, there was an increase in the expression of several proteins involved in apoptosis-related pathways in CLL-B-cells, and that, regardless of the EVs source. Among them, BCL-2, whose involvement in CLL has already been extensively described [48] and whose therapeutic targeting with mimetic compounds has already shown to be efficient for some CLL patients [49, 50]. There was also an increase in IGFBP-2 levels, while there is no direct evidence of the relationship between IGFBP-2 and CLL, it is highly expressed in certain human acute leukemia cells, and it supports the activity of hematopoietic stem cells (HSCs) [51]. Therefore, it is possible that IGFBP-2 may play a role in the development and progression of CLL, but more research is needed to confirm this hypothesis. Conversely to IGFBP-2, the relationship between CD40 and CLL is well documented. For example, CLL cells strongly depend on interactions with bystander T cells and monocyte-derived cells (MDCs) within the lymph node (LN) microenvironment for their survival and resistance to therapy [52]. CD40 is a critical stimulatory receptor on antigen-presenting cells of the immune system. CD40-mediated activation of B cells has been shown to initiate a variety of signals in B cells including the activation of MAP kinases and NF-κB [53] which are strongly linked to pathogenesis [54, 55] and treatment resistance [56]. An increased level of p53 protein was also observed, it has already been linked to an advanced clinical stage, more progressive forms of the disease, poor response to therapy and an overall lower survival [36]. Furthermore, a study by Samuel et al. describes that a 30- to 40-fold induction of wild-type p53 protein was observed in 50 distinct human CLL patients tested, without the induction of either cell-cycle arrest or apoptosis, however, the mRNA levels for p53 did not increase, indicating that its elevation occurred post-transcriptionally [57]. We would nevertheless like to point out that in these experiments, the proper control (EVs from healthy monocytes/macrophages) is missing due to lack of biological material.

To assess the potential impact of NLCs EVs on CLL-B-cells gene expression, we evaluated mRNA expression levels of CLL-B-cells treated or not with NLCs EVs through RNAseq analysis. We found that EVs treatment induced changes in the expression of various genes, some already known for their involvement in pathogenesis. Among the upregulated genes, we notably found *RGS1* and *DAD1*. *RGS1* has been found to be upregulated in several cancers, it is an unfavorable prognostic marker in renal and stomach cancer [58], high immunohistochemical expression of *RGS1* has also been linked to poor overall survival in diffuse large B cell lymphoma (DLBCL) patients [59]. Furthermore, a study aiming at identifying discriminatory and differentially expressed genes related to various cellular processes in CLL highlighted *RGS1* as one of the genes with potential significance in predicting IgVH mutational status [60]. In multiple cancers, *RGS1* has been found to promote T-cell exhaustion [61], in breast cancer for instance, its upregulation reduced trafficking of anti-tumor lymphocytes to tumors and was associated with shorter survival of patients [62]. As for *DAD1*, it is a negative regulator of cell death associated with the endoplasmic reticulum cell death pathway [63] that has been linked to tumorigenesis in various cancers [64]. It was found to be upregulated in CLL and has been associated with poorer overall survival [65].

Regarding the down-regulated genes, *SPEN*, a transcriptional repressor, has been identified as a tumor suppressor that is able to regulate cell proliferation, tumor growth, and survival in hormone-dependent breast cancers [66]. In mantle cell lymphoma (MCL), an incurable subtype of non-Hodgkin lymphoma, *SPEN* was found to be one of the genes with the highest mutational frequency difference from baseline samples (samples taken at the time of diagnosis) to samples taken at more advanced stages of the disease, suggesting its potential role in disease progression [67]. Another article highlights the relationship between *SPEN* mutations and CLL, the study reveals that *SPEN* mutations are present in 4% of high-risk CLL cases, impacting the *NOTCH1* signaling pathway through the derepression of *NOTCH1* target genes such as *HES1*, *DTX1*, and *MYC* [68].

*AHNAK* was also found to be down-regulated in the samples treated with NLCs EVs. AHNAK family members are involved in the regulation of various biological functions, such as calcium channel modulation and membrane repair but have also been associated to the development of several cancers [69]. Cai et al. established that *AHNAK* was downregulated in ovarian cancer and showed that elevated *AHNAK* expression in ovarian cancer cell lines repressed cell growth and metastasis both in vitro and in vivo notably through the inhibition of the WNT/β-catenin signaling pathway [70]. In a study examining the role of formin-like 1 (FMNL1) in primary hematopoietic cells derived from CLL patients, AHNAK was identified as one of his binding partners. FMNL1 is a formin-related protein highly expressed in hematopoietic cells and also overexpressed in leukemias, its interaction with AHNAK induced the localization of the latter at the cell membrane thereby suggesting a potential role of both of these actors in pathogenesis [71].

Finally, among those down-regulated genes, we found *AMER1*, APC (adenomatous polyposis coli) membrane recruitment protein 1. In gastric cancer, through its interaction with APC, it negatively regulates canonical WNT signaling, therefore acting as a tumor suppressor similarly to *AHNAK* [72, 73].

These results show that NLCs are in fact able to modulate the expression of genes potentially involved in pathogenesis in leukemic cells through the production of EVs. However, they remain preliminary given the small amount of analyzed samples and need further investigation to precisely identify the actors involved. As they are, they solely underline the ability of NLCs to induce gene expression modifications in CLL-B-cells using EVs as a communication tool, therefore reinforcing the point that EVs are key players within the tumor microenvironment.

Upon the observation of the changes induced by EVs, we pondered their potential content. To investigate it, we performed a whole proteome analysis of EVs derived from M2-THP1 cells. Results highlighted the presence of many proteins involved in pathways critical to pathogenesis. Among them, pathways relating to cell death, transcriptional regulation, and various signaling pathways linked to oncogenesis. This analysis offers valuable insight regarding the protein cargo of EVs, but it remains a study of an in vitro model and not actual patient-derived NLCs. It would be of interest to perform the same analysis on NLCs EVs if we are ever able to obtain them in sufficient quantities. First because they are a more relevant model but also because it would serve as a comparison to further validate and underline the relevance of M2-THP1 cells as an in vitro model for NLCs.

Finally, in addition to their effect on CLL-B-cells apoptosis and proliferation rate, we tested the effect of EVs on treatment resistance. EVs from NLCs increased the resistance of CLL-B-cells to Ibrutinib in vitro. It is however important to note that in vitro analyses remain preliminary, further analyses are needed notably regarding the effect on the cell cycle and these results should be confirmed in vivo, especially when it comes to complex multifactorial processes such as treatment resistance.

Overall, these results show that EVs from the microenvironment, and not only EVs from malignant B-cells [13], are able to modulate their recipient’s cell activity, sending oncogenic signals to promote leukemic cell aggressiveness. NLCs had previously been identified as key actors in the TME [32] but they had never been shown to transfer information through EVs before. This work underlines the potential of NLCs as therapeutic targets and the pertinence of EVs as biomarkers in this pathology. We would like to finish by pointing out that the generation of purified NLCs in vitro can be challenging and that, by definition, NLCs are dependent on the presence of adjacent B CLL cells. Leaving them in culture medium without B CLL for 48 h to retrieve their EVs could potentially affect the cells, therefore making them more or less different from their in vivo counterparts. However, this paper still constitutes a proof-of-concept demonstrating this mode of inter-cellular communication, which opens the door to future studies on larger cohorts that will take better account of the heterogeneity of this pathology and validate the functional importance of EVs in communication between NLCs and malignant B-cells.

### Supplementary information

Supplementary data

Supplementary Table 3

Supplementary Table 4

Original data files

qPCR raw data

## Data Availability

Data will be made available upon request to the corresponding author.
