# Supplementary material for: Extracellular vesicles from type-2 macrophages increase the survival of chronic lymphocytic leukemia cells ex vivo
Source: Cancer Gene Ther. 2024 Jun 25;31(8):1164–76. doi: 10.1038/s41417-024-00802-7 (PMC11327105; doi:10.1038/s41417-024-00802-7)
Supplement: Supplementary file 1 — Supplementary data [file 41417_2024_802_MOESM1_ESM.pdf]

Supplementary data

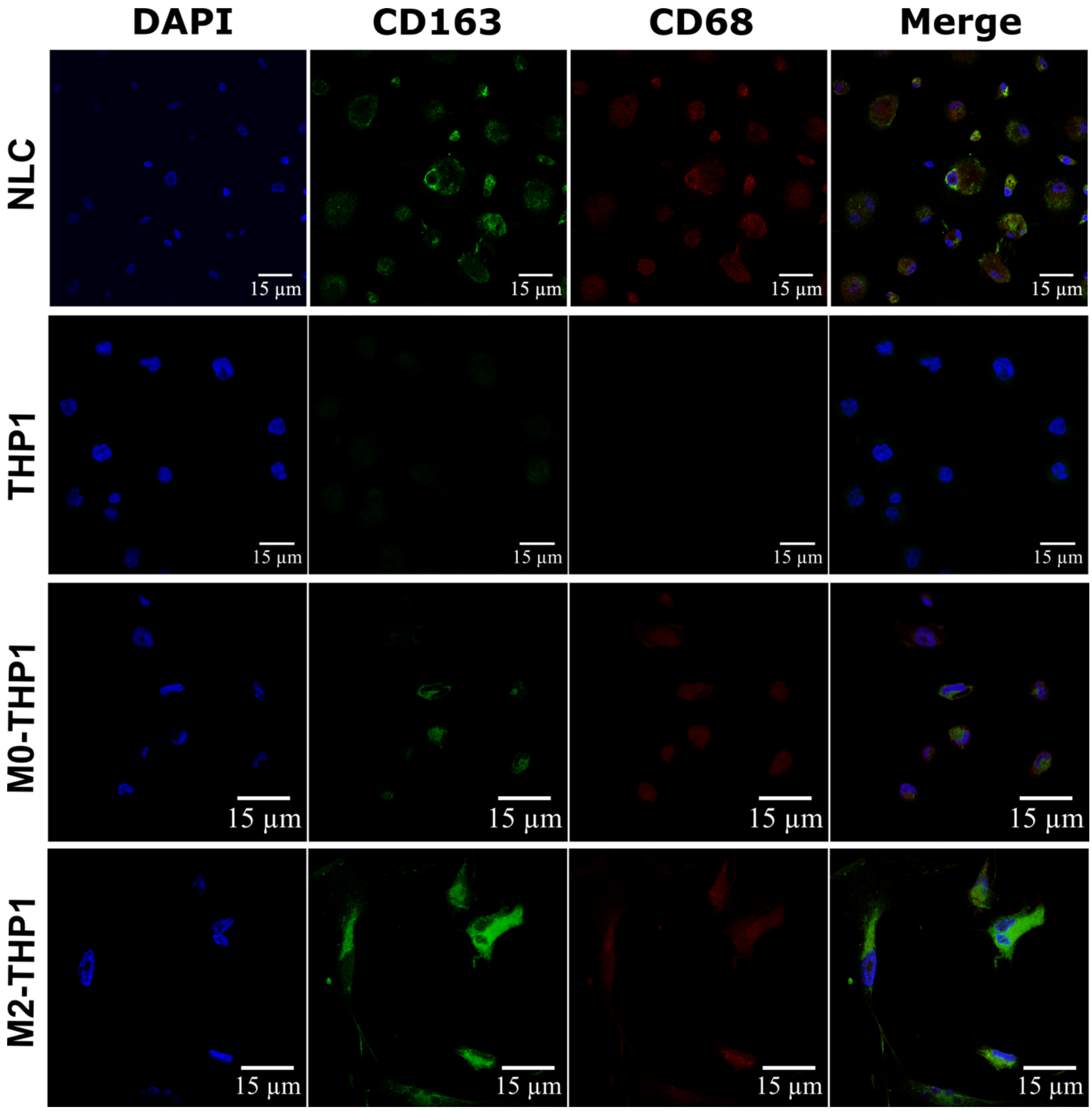

<u>Supplementary Figure 1</u>: **Control of M2-subtype macrophages markers expression in patient-derived NLCs and polarized THP1 cells**. Cells were seeded on coverslips within 12-well plates. Cells on the coverslips were then washed, fixed and blocked before being incubated with fluorochrome-coupled primary antibodies (anti-CD68 Alexa Fluor 647 #51-0689-42, Invitrogen & anti-CD163 Alexa Fluor 488 #568188, BD Biosciences) at a dilution of 1/100 for 1h at 4°C in the dark. They were then washed and mounted on slides using DAPI containing mounting medium. Slides were observed using LSM880 confocal microscope (Zeiss) at x40 magnification and data analyzed using Zeiss ZEN software and ImageJ.

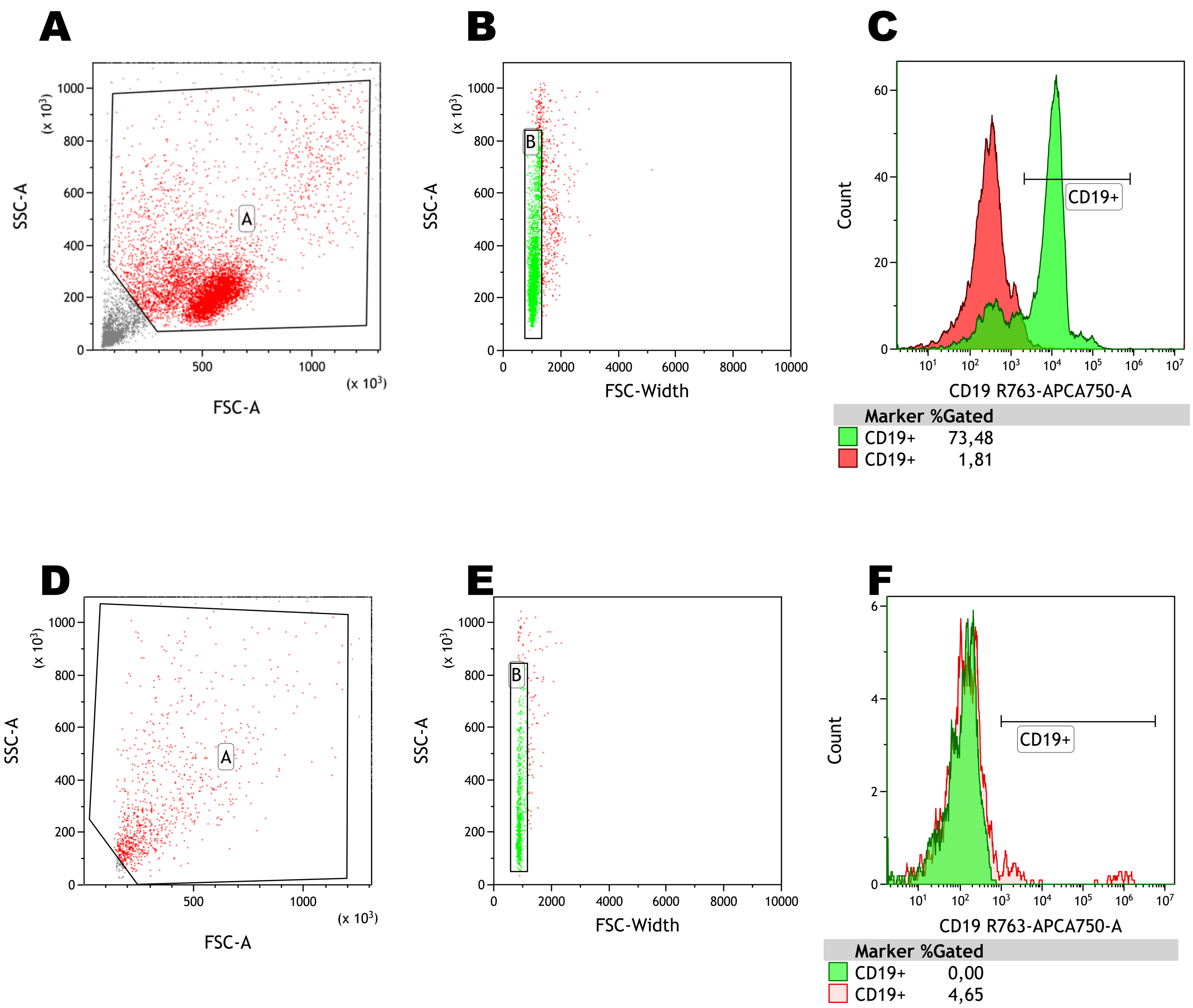

<u>Supplementary Figure 2</u>: Measurement of CD19-positive cells after NLC differentiation protocol. PBMCs isolated from CLL patients (5x10^6 cells) were incubated for two weeks in 5 mL of RPMI medium in a 6-well plate. PBMCs were collected and labeled with anti-CD19 antibodies, then analyzed by FACS. Figure (**A**) represents ungated PBMCs analyzed based on FSC and SSC. Cells selected within gate A were further analyzed for FSC-W and SSC to exclude cell aggregates (**B**). In Figure (**C**), the gated cells from B were analyzed for CD19 labeling. Before washing, approximately 73% of cells in the culture supernatant were CD19+ cells (B cells). The red curve represents isotypic labeling. Figures (**D**), (**E**), and (**F**) correspond to the analysis of NLC after B-cells removal by vigorous washing. After two weeks of PBMC incubation, cells in the supernatant were removed, and adherent NLCs were thoroughly washed three times with culture medium. After washing, NLCs were recovered *via* trypsin/EDTA treatment, and cells were labeled with an anti-CD19 antibody before FACS analysis. Figure (**D**) illustrates FSC/SSC analysis of unlabeled cells (predominantly NLC). Cells selected within

gate A were then analyzed based on FSC-W/SSC to exclude aggregates, and the selected cells within gate B were analyzed for CD19 labeling. As observed, no CD19-positive cells were detected. The red curve corresponds to isotypic labeling.

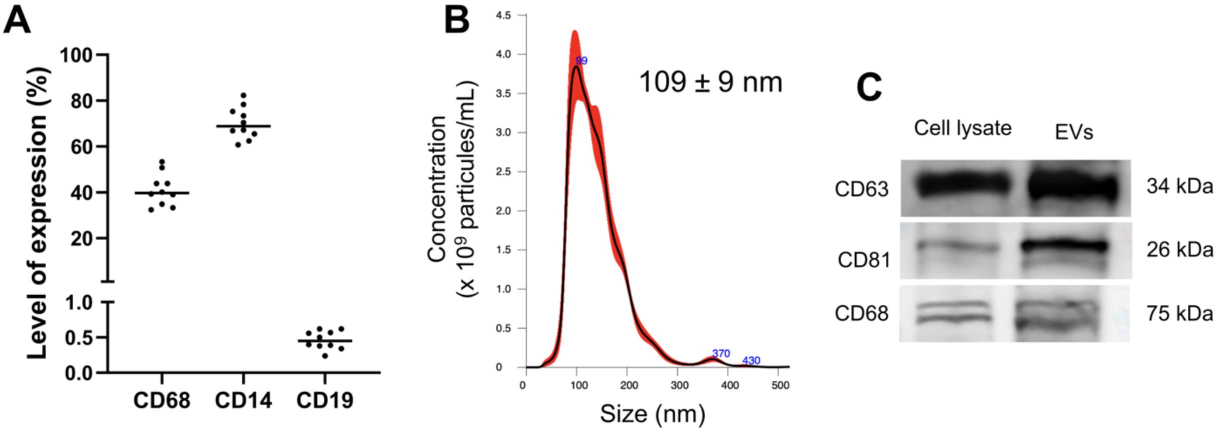

<u>Supplementary Figure 3</u>: Characterization of healthy donors' monocytes/macrophages and their EVs. **(A)** Monocytes isolated from healthy donors' blood were harvested and stained with anti-CD68 and anti-CD14 fluorochrome-coupled antibodies to check for macrophage/monocyte markers, as well as anti-CD19 fluorochrome-coupled antibody to check for any possible B cell contamination. They were then fixed and analyzed by flow cytometry. The presented graph represents the observed levels of expression, the line represents the mean. **(B)** Evaluation of particle size by Nanoparticle Tracking Analysis (NTA) using the NanoSight NS3000 (Malvern Panalytical) of EVs from healthy monocytes/macrophages. The numerical value is the mode (nm) +/- standard error. **(C)** Western blot analysis of EVs markers CD63 and CD81 and macrophage marker CD68.

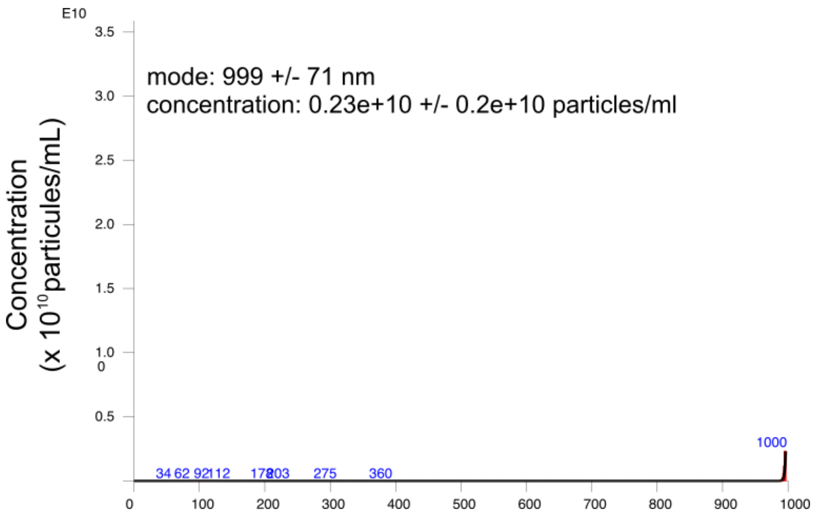

<u>Supplementary Figure 4</u>: Evaluation of the presence of EVs in the 'exo-free' medium. Evaluation of particle size and concentration in the 'exo-free' medium by Nanoparticle Tracking Analysis (NTA) using the NanoSight NS3000 (Malvern Panalytical). Mode (nm) and concentration are written +/- standard error.

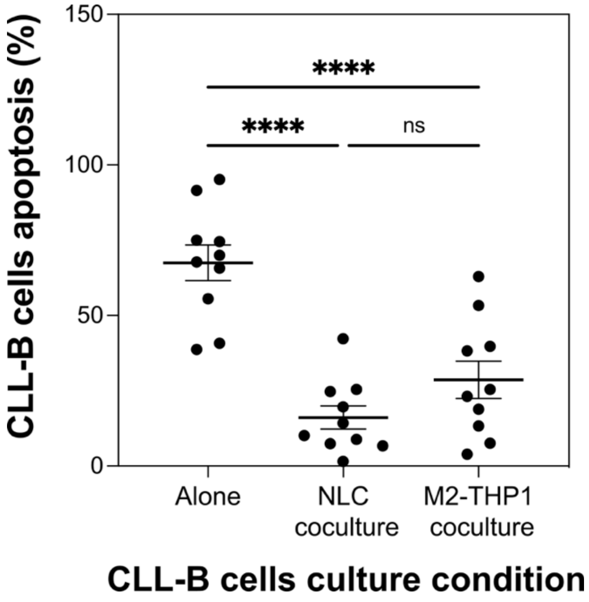

<u>Supplementary Figure 5</u>: M2-THP1 cells and NLCs protect CLL patients B cells from apoptosis in co-culture. Annexin V / IP labelling was used. 500 000 CLL-B-cells (before or after treatment) were resuspended in 100 µL of PBS/Ca^{2+} and incubated with 5 µL of Annexin V-FITC (90 µg/ml) in the dark on ice for 15 minutes. Then, 400 µL of PBS/Ca^{2+} and 5 µL of IP (0.5 mg/ml) were added just before acquisition on the FACSCalibur flow cytometer (BD Biosciences).

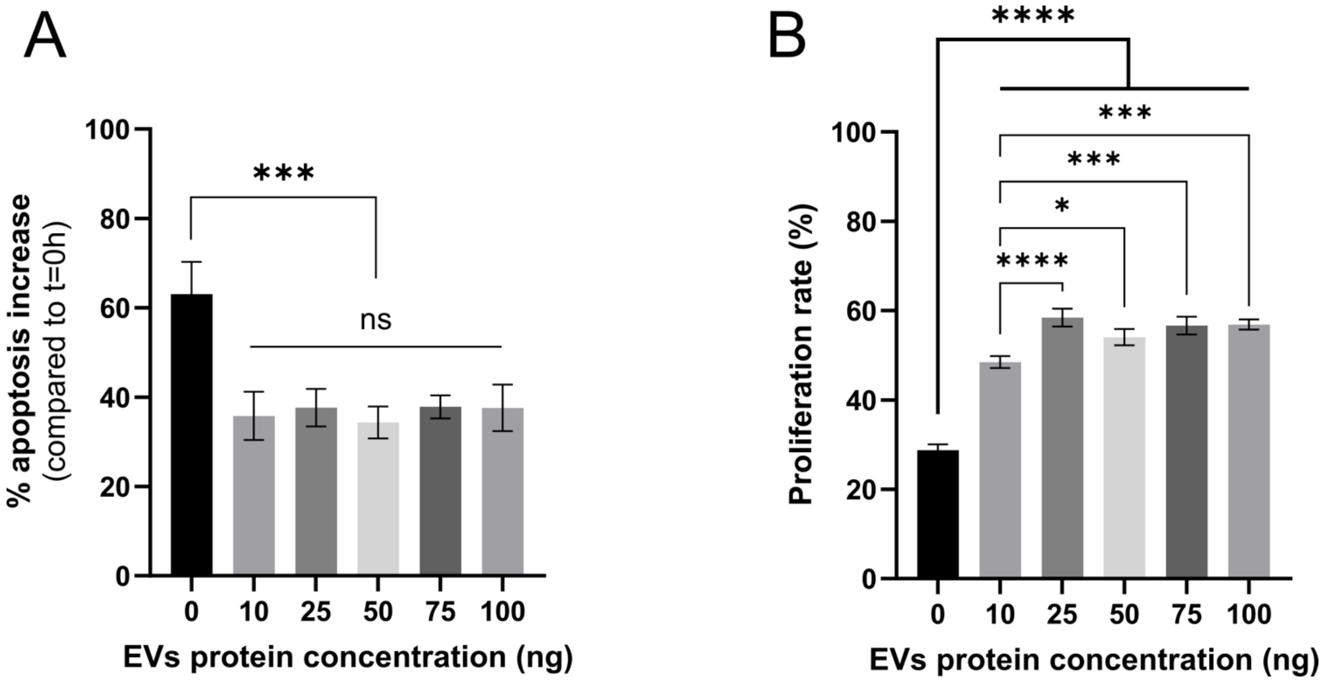

<u>Supplementary Figure 6</u>: Test of an EVs protein concentration range. 25 000 CLL-B cells (patient #1) were seeded in a 96-well plate and treated with a range of M2-THP1 EVs protein concentrations to determine an optimal dose. 25 ng was selected for all the following experiments (1.10^{-3} ng of EVs/cell). Experiments were conducted using the Incucyte live-cell imaging device, cells were marked with Annexin V (Sartorius) and fluorescence was monitored for 48h. The percentage of cells in apoptosis and the cell count were determined using the Incucyte base analysis software. Results are presented as mean SEM. **(A)** The percentage of apoptosis at t=0h was substracted to the percentage of apoptosis at 48h for each treatment condition. The graph plots the result of this substraction which reflects the increase of CLL-B-cells apoptosis over a period of 48h. **(B)** The proliferation rate (increase rate of the cell count) was determined over a period of 48h.

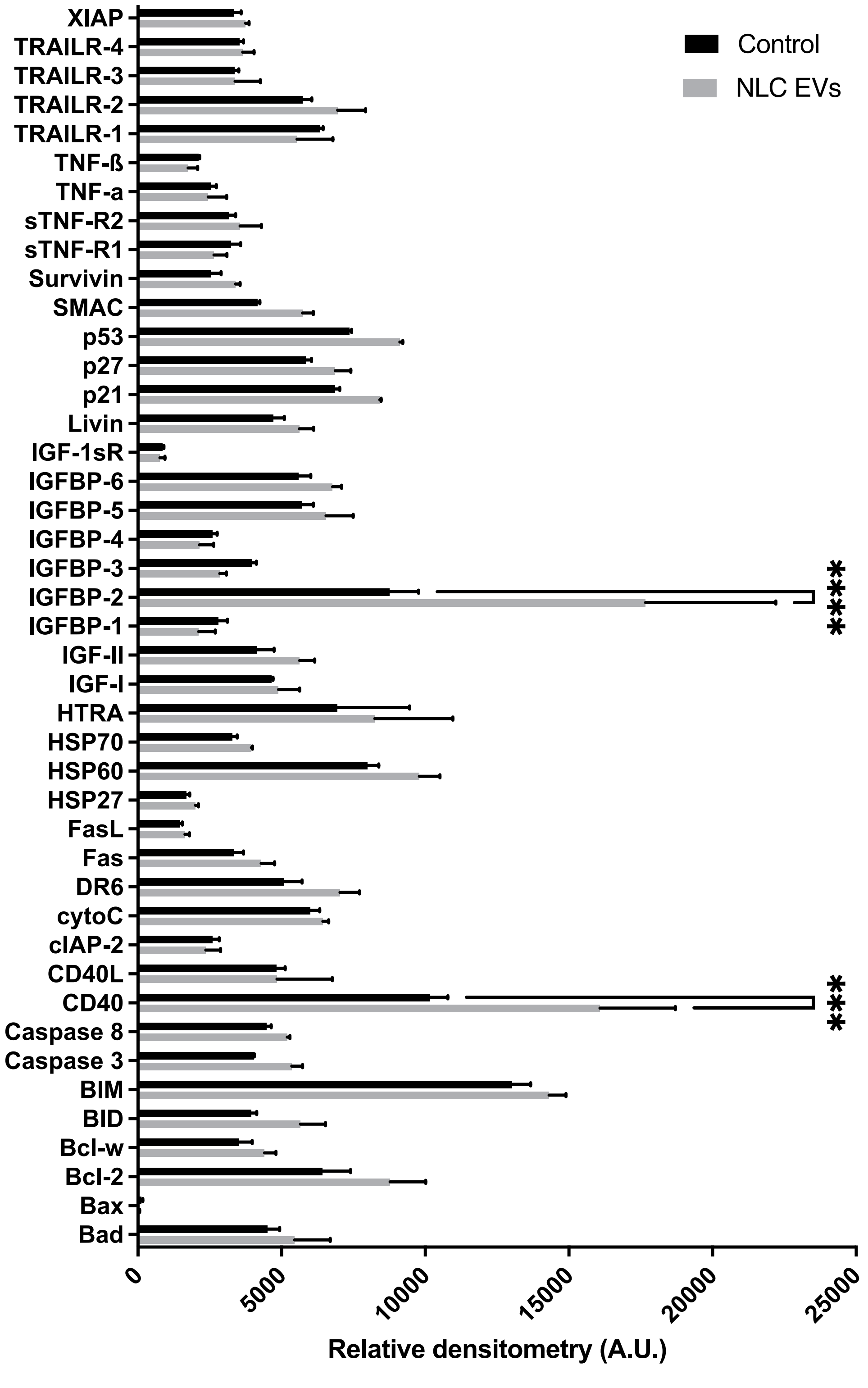

<u>Supplementary Figure 7</u>: **Proteome profiler array**. CLL-B cells from 2 patients (#4 and 10) were treated with EVs from NLCs and their protein extracts were incubated with an antibody array membrane (Abcam) to explore apoptosis-related pathways. Results are presented as mean □ SD.

Supplementary Table 1: Characteristics of the patients included in the study.

Patient ID number	Binet stage	cytogenetics	TP53	IGHV mutation	Total prognosis (cytogenetics and/or mutations)
1	C	Favorable del13q	unmutated	mutated, favorable	not adverse
2	B	Adverse del13q del17p	mutated and deleted (R181C ; R342*)	unmutated, adverse	adverse
3	A	ND	ND	ND	ND
4	B	Adverse del11q del6q	unmutated	unmutated, adverse	adverse
5	A	ND	ND	ND	ND
6	C	Adverse +12, del14q	mutated (R273C ; G244D)	unmutated, adverse	adverse
7	C	adverse del17p (TP53) del13q	mutated and deleted (C135Y)	mutated, favorable	adverse
8	C	Favorable del13q	unmutated	unmutated, adverse	adverse
9	C	Intermediate +12	mutated	unmutated, adverse	adverse
10	A	Favorable del13q	ND	mutated, favorable	not adverse
11	C	Adverse del6q, del13q, del17p	mutated	unmutated, adverse	adverse
12	B	Adverse Complex >3	mutated	unmutated, adverse	adverse
13	C	Adverse Complex >5	ND	unmutated, adverse	ND
14	C	Intermediate del14q	unmutated	unmutated, adverse	intermediate
15	C	Adverse Complex >5	unmutated	unmutated, adverse	adverse
16	C	Favorable del13q	unmutated	mutated, subset 2	intermediate

Supplementary Table 2: List of antibodies used

Target	Host species	Reference	Manufacturer	Application
CD9	rabbit	13403S	Cell signaling	WB
CD63	mouse	cbl50053	Merck	WB
CD81	mouse	sc-166029	Santa cruz	WB
CD68	mouse	sc-20060	Santa cruz	WB
IGFBP2	mouse	16848715	Proteintech	WB
CD40	mouse	16889295	Proteintech	WB
p53	rabbit	16871435	Proteintech	WB

Bcl-2	rabbit	16810694	Proteintech	WB
CD19 APC-H7	IgG1,κ mouse	560727	BDbioscience	Flow cytometry
CD14-ECD	IgG2a mouse	B92391	Beckman Coulter	Flow cytometry
CD68-FITC	IgG2b, κ mouse	562117	BDbioscience	Flow cytometry
APC-H7 Mouse IgG1, κ Isotype Control	IgG1,κ mouse	561427	Beckman Coulter	Flow cytometry
IgG2a Mouse-ECD, Isotype control	IgG2a mouse	A09144	BDbioscience	Flow cytometry
FITC Mouse IgG2b, κ Isotype Control	IgG2b, κ mouse	555057	BDbioscience	Flow cytometry
