# Supplementary material for: Extracellular vesicles from type-2 macrophages increase the survival of chronic lymphocytic leukemia cells ex vivo
Source: Cancer Gene Ther. 2024 Jun 25;31(8):1164–76. doi: 10.1038/s41417-024-00802-7 (PMC11327105; doi:10.1038/s41417-024-00802-7)
Supplement: Supplementary file 2 — Supplementary Table 3 [file 41417_2024_802_MOESM2_ESM.docx]

**Supplementary Table 3:** List of differentially expressed genes (DEGs) in EVs-treated cells (n=3) as compared to control untreated cells (n=3) from paired analysis on RNASeq data, with absolute fold change superior to 1.5 and p adjusted inferior to 0.1. Red and green colors indicate up- and down-regulated genes in EVs-treated cells in comparison to control untreated cells, respectively.

| **Ensembl ID** | **log2 Fold Change** | **p value** | **p adjusted** | **Gene Symbol** | **Gene Name** | **Gene ID** | **Gene Type** |
| --- | --- | --- | --- | --- | --- | --- | --- |
| ENSG00000167978 | **-0,78** | 2,02E-07 | 0,003 | **SRRM2** | serine/arginine repetitive matrix 2 | 23524 | protein coding |
| ENSG00000065526 | **-0,72** | 4,42E-06 | 0,010 | **SPEN** | spen family transcriptional repressor | 23013 | protein coding |
| ENSG00000090975 | **-0,63** | 1,96E-06 | 0,010 | **PITPNM2** | phosphatidylinositol transfer protein membrane associated 2 | 57605 | protein coding |
| ENSG00000237550 | **1,02** | 3,09E-06 | 0,010 | **RPL9P8** | ribosomal protein L9 pseudogene 8 | 254948 | transcribed processed pseudogene |
| ENSG00000251143 | **-0,81** | 4,45E-06 | 0,010 | **LOC100128494** | uncharacterized LOC100128494 | 100128494 | lncRNA |
| ENSG00000167615 | **-0,74** | 1,64E-05 | 0,032 | **LENG8** | leukocyte receptor cluster member 8 | 114823 | protein coding |
| ENSG00000111678 | **0,66** | 2,44E-05 | 0,036 | **C12orf57** | chromosome 12 open reading frame 57 | 113246 | protein coding |
| ENSG00000124942 | **-0,61** | 2,68E-05 | 0,036 | **AHNAK** | AHNAK nucleoprotein | 79026 | protein coding |
| ENSG00000204681 | **-0,61** | 2,29E-05 | 0,036 | **GABBR1** | gamma-aminobutyric acid type B receptor subunit 1 | 2550 | protein coding |
| ENSG00000272173 | **0,64** | 3,22E-05 | 0,040 | **NA** | NA | NA | lncRNA |
| ENSG00000127481 | **-0,69** | 3,90E-05 | 0,042 | **UBR4** | ubiquitin protein ligase E3 component n-recognin 4 | 23352 | protein coding |
| ENSG00000038382 | **-0,66** | 6,12E-05 | 0,050 | **TRIO** | trio Rho guanine nucleotide exchange factor | 7204 | protein coding |
| ENSG00000112695 | **0,80** | 6,24E-05 | 0,050 | **COX7A2** | cytochrome c oxidase subunit 7A2 | 1347 | protein coding |
| ENSG00000168288 | **0,72** | 7,67E-05 | 0,055 | **MMADHC** | metabolism of cobalamin associated D | 27249 | protein coding |
| ENSG00000181350 | **0,68** | 9,04E-05 | 0,058 | **LRRC75A** | leucine rich repeat containing 75A | 388341 | protein coding |
| ENSG00000258727 | **-0,61** | 8,85E-05 | 0,058 | **AP1G2-AS1** | AP1G2 antisense RNA 1 | 102724814 | lncRNA |
| ENSG00000084112 | **-0,63** | 1,79E-04 | 0,067 | **SSH1** | slingshot protein phosphatase 1 | 54434 | protein coding |
| ENSG00000115350 | **0,82** | 1,31E-04 | 0,067 | **POLE4** | DNA polymerase epsilon 4, accessory subunit | 56655 | protein coding |
| ENSG00000129562 | **0,67** | 1,60E-04 | 0,067 | **DAD1** | defender against cell death 1 | 1603 | protein coding |
| ENSG00000173821 | **-0,61** | 1,34E-04 | 0,067 | **RNF213** | ring finger protein 213 | 57674 | protein coding |
| ENSG00000174173 | **0,70** | 1,74E-04 | 0,067 | **TRMT10C** | tRNA methyltransferase 10C, mitochondrial RNase P subunit | 54931 | protein coding |
| ENSG00000175602 | **0,61** | 1,67E-04 | 0,067 | **CCDC85B** | coiled-coil domain containing 85B | 11007 | protein coding |
| ENSG00000184675 | **-0,86** | 1,78E-04 | 0,067 | **AMER1** | APC membrane recruitment protein 1 | 139285 | protein coding |
| ENSG00000226890 | **-2,06** | 1,42E-04 | 0,067 | **NA** | NA | NA | lncRNA |
| ENSG00000255026 | **-0,93** | 1,09E-04 | 0,067 | **NA** | NA | NA | lncRNA |
| ENSG00000259589 | **-2,01** | 1,34E-04 | 0,067 | **NA** | NA | NA | lncRNA |
| ENSG00000090104 | **1,12** | 2,57E-04 | 0,079 | **RGS1** | regulator of G protein signaling 1 | 5996 | protein coding |
| ENSG00000184677 | **-0,59** | 2,89E-04 | 0,080 | **ZBTB40** | zinc finger and BTB domain containing 40 | 9923 | protein coding |
| ENSG00000184983 | **0,70** | 2,87E-04 | 0,080 | **NDUFA6** | NADH:ubiquinone oxidoreductase subunit A6 | 4700 | protein coding |
| ENSG00000125356 | **0,80** | 3,15E-04 | 0,084 | **NDUFA1** | NADH:ubiquinone oxidoreductase subunit A1 | 4694 | protein coding |
| ENSG00000096070 | **-0,72** | 3,67E-04 | 0,087 | **BRPF3** | bromodomain and PHD finger containing 3 | 27154 | protein coding |
| ENSG00000115128 | **0,77** | 3,75E-04 | 0,087 | **SF3B6** | splicing factor 3b subunit 6 | 51639 | protein coding |
| ENSG00000181061 | **0,83** | 4,13E-04 | 0,095 | **HIGD1A** | HIG1 hypoxia inducible domain family member 1A | 25994 | protein coding |
| ENSG00000242294 | **-1,42** | 4,33E-04 | 0,096 | **STAG3L5P** | STAG3 cohesin complex component like 5, pseudogene | NA | transcribed unprocessed pseudogene |
| ENSG00000279933 | **-0,64** | 4,39E-04 | 0,096 | **NA** | NA | NA | TEC |
| ENSG00000114520 | **0,63** | 4,70E-04 | 0,097 | **SNX4** | sorting nexin 4 | 8723 | protein coding |
| ENSG00000126860 | **0,71** | 4,75E-04 | 0,097 | **EVI2A** | ecotropic viral integration site 2A | 2123 | protein coding |
| ENSG00000188157 | **-1,75** | 4,84E-04 | 0,098 | **AGRN** | agrin | 375790 | protein coding |
| ENSG00000196187 | **-0,60** | 4,95E-04 | 0,098 | **TMEM63A** | transmembrane protein 63A | 9725 | protein coding |
