# Supplementary material for: Extracellular vesicles from type-2 macrophages increase the survival of chronic lymphocytic leukemia cells ex vivo
Source: Cancer Gene Ther. 2024 Jun 25;31(8):1164–76. doi: 10.1038/s41417-024-00802-7 (PMC11327105; doi:10.1038/s41417-024-00802-7)

Flow cytometry – Healthy donors monocytes/macrophages

HEALTHY DONOR MONOCYTES - 1

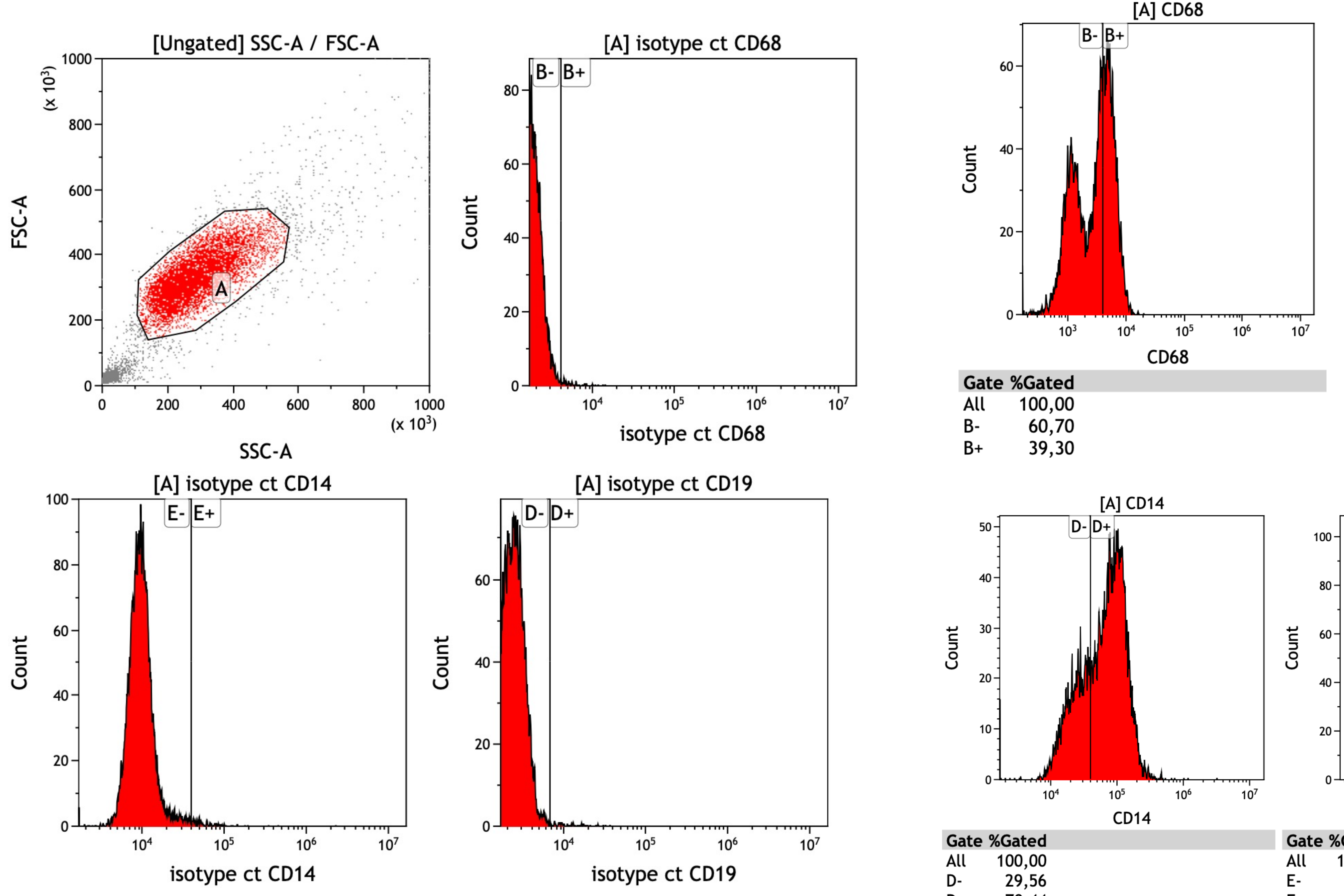

Gate	%Gated
All	100,00
B-	60,70
B+	39,30

Gate	%Gated
All	100,00
D-	29,56
D+	70,44

Gate	%Gated
All	100,00
E-	99,76
E+	0,24

HEALTHY DONOR MONOCYTES - 2

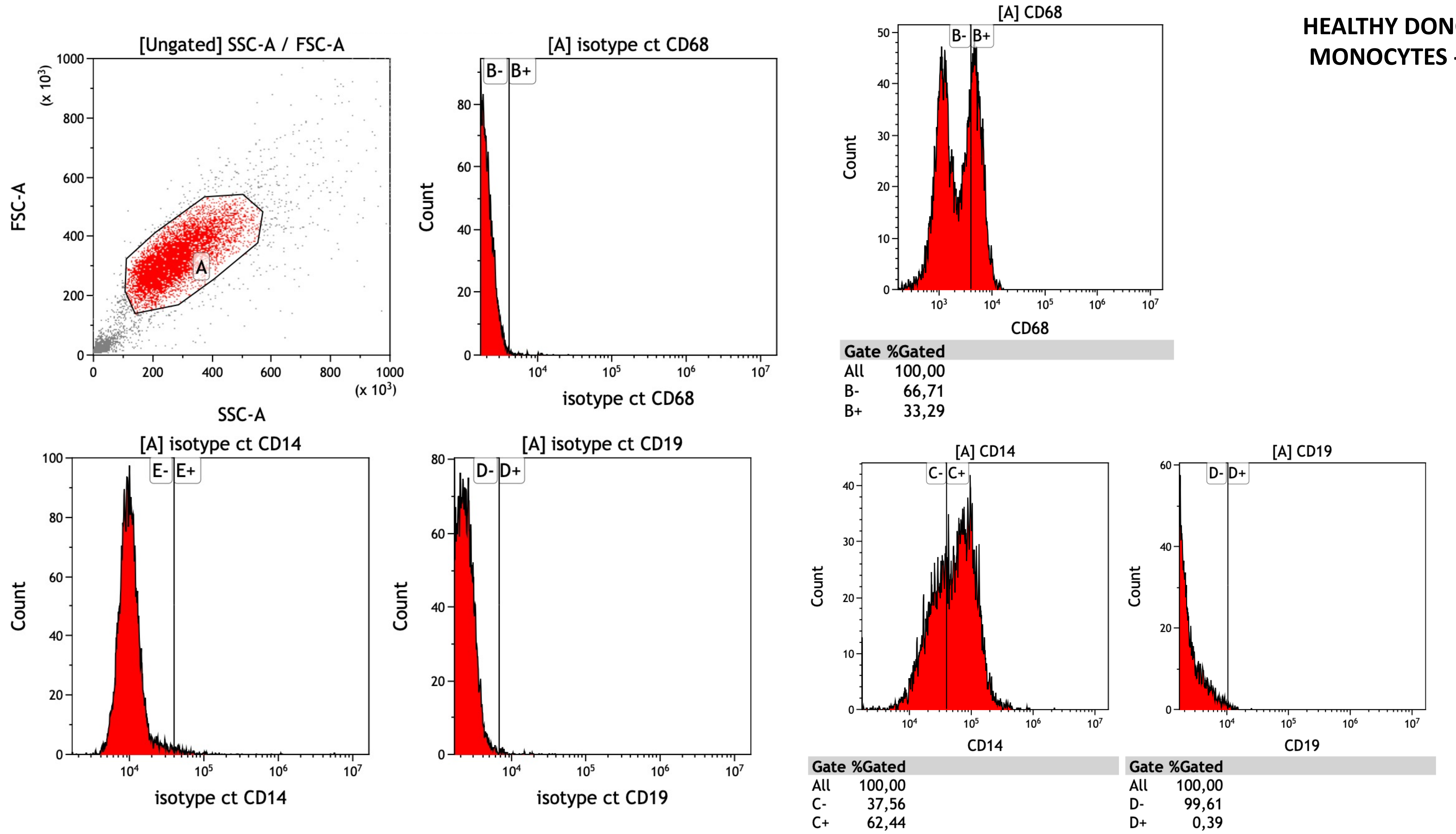

Gate	%Gated
All	100,00
B-	66,71
B+	33,29

Gate	%Gated
All	100,00
C-	37,56
C+	62,44

Gate	%Gated
All	100,00
D-	99,61
D+	0,39

HEALTHY DONOR MONOCYTES - 3

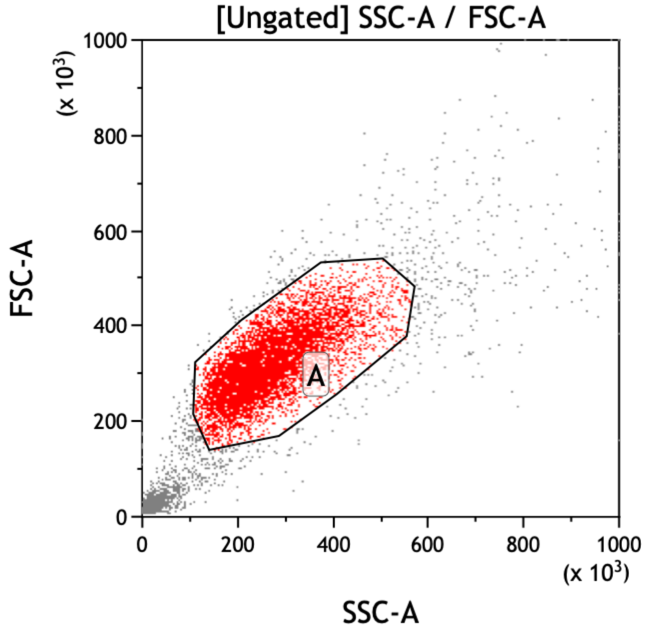

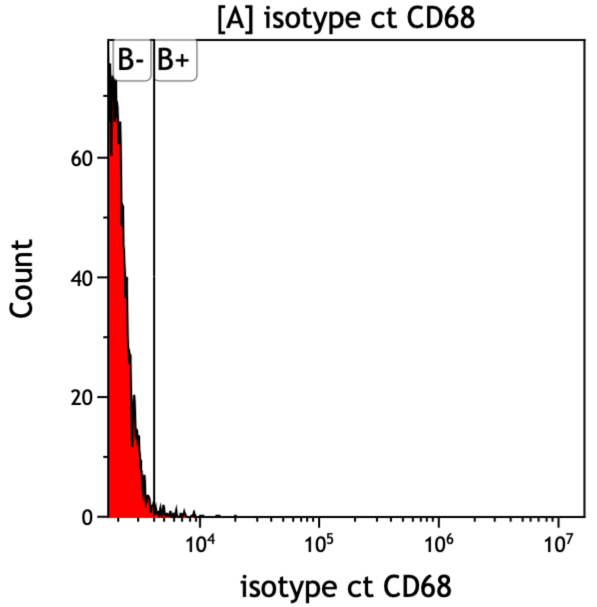

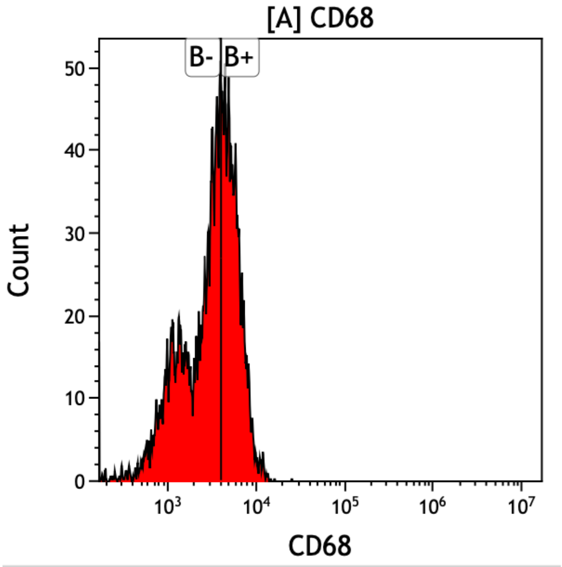

Gate	%Gated
All	100,00
B-	59,96
B+	40,04

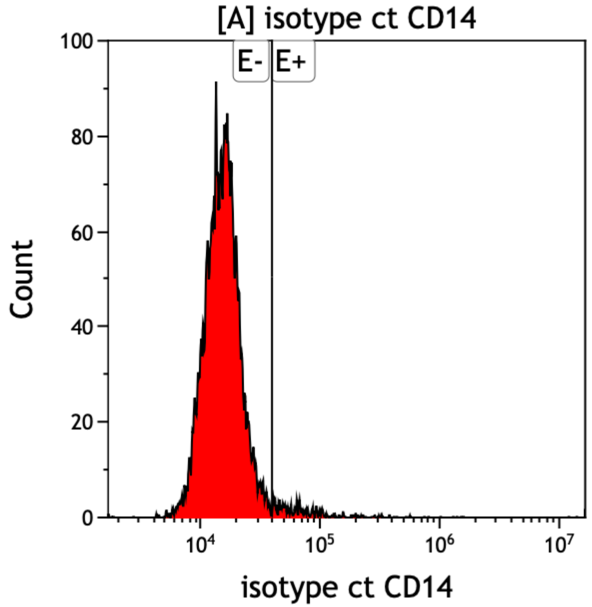

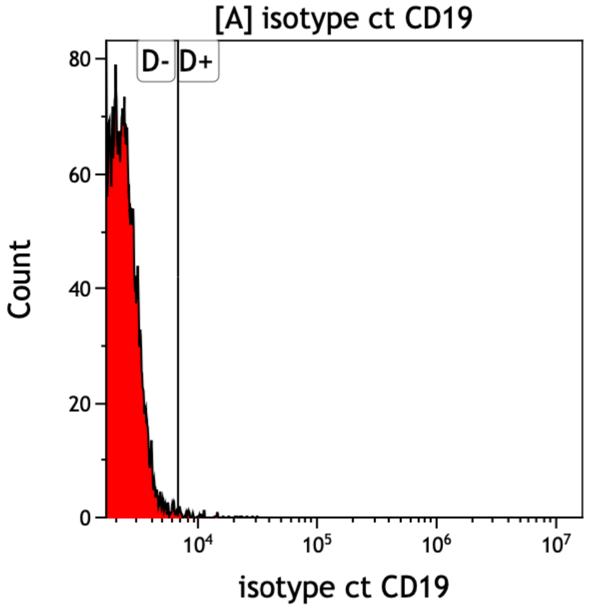

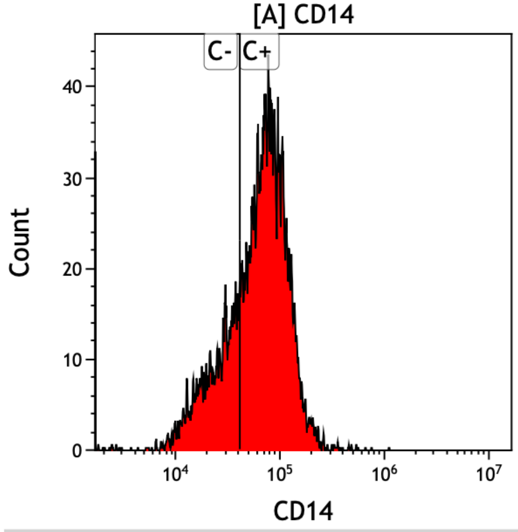

Gate	%Gated
All	100,00
C-	26,62
C+	73,38

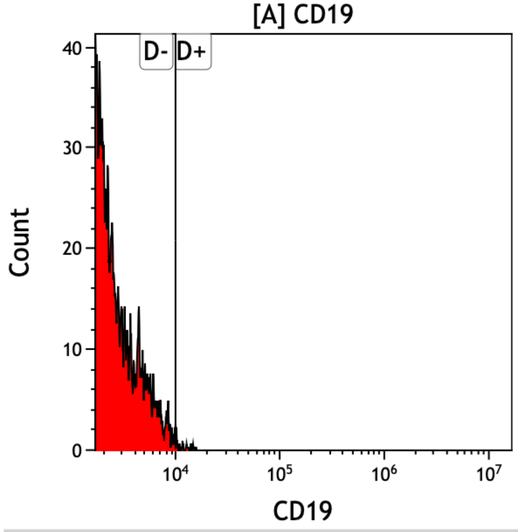

Gate	%Gated
All	100,00
D-	99,60
D+	0,40

HEALTHY DONOR MONOCYTES - 4

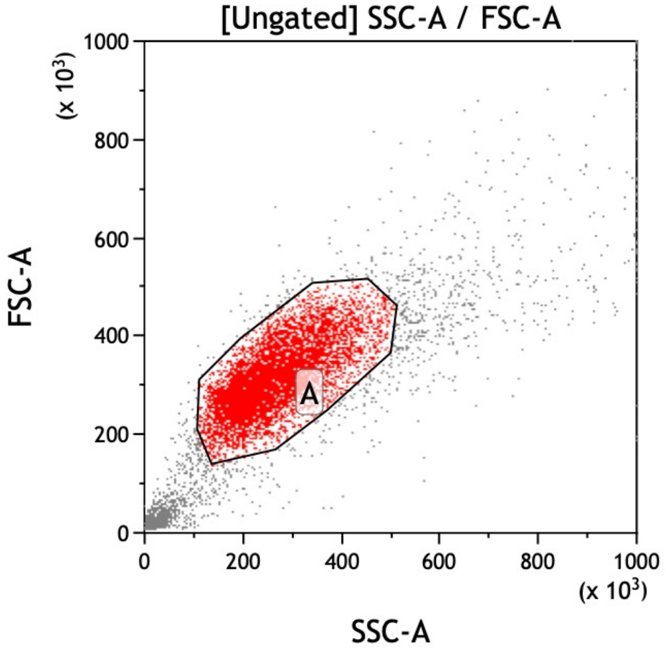

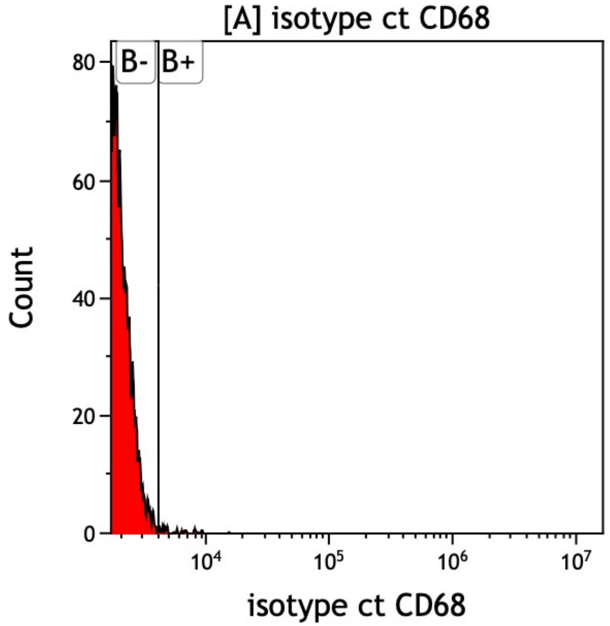

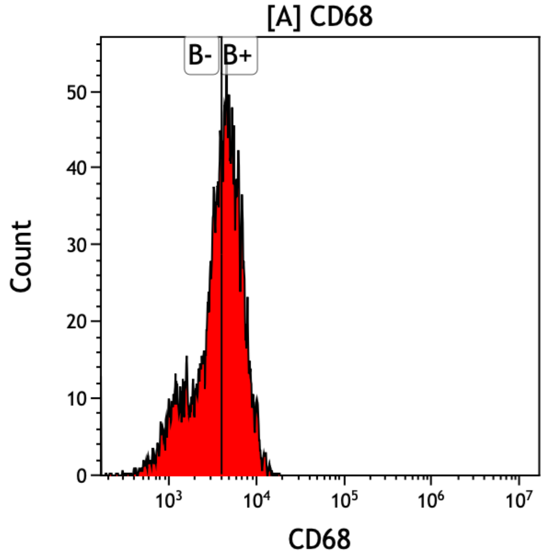

Gate	%Gated
All	100,00
B-	46,65
B+	53,35

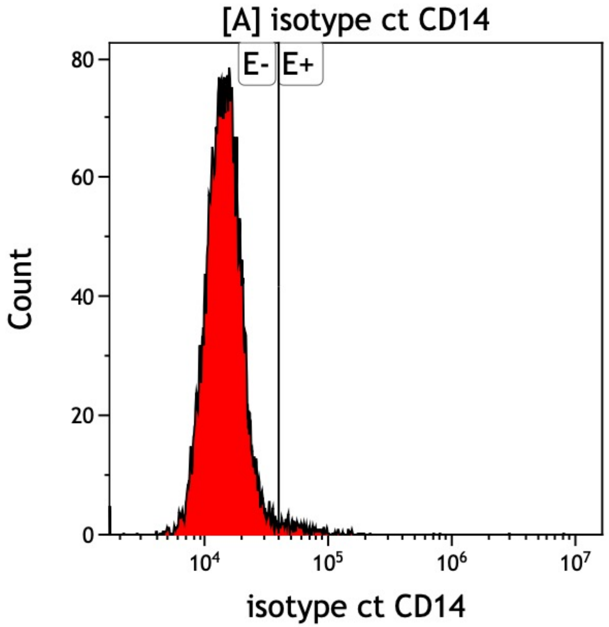

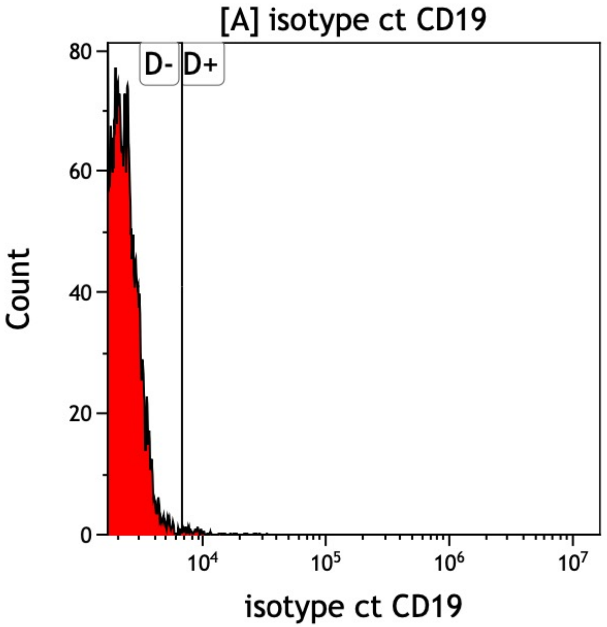

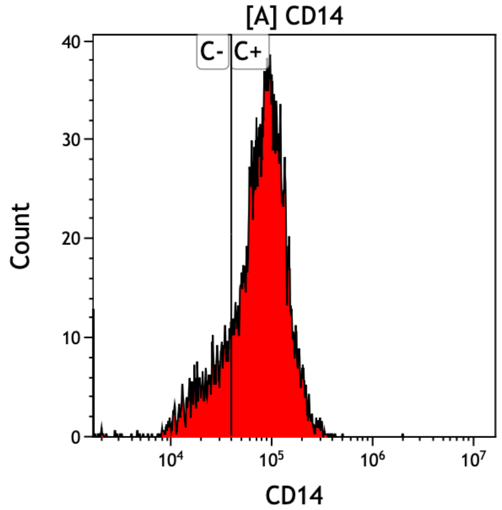

Gate	%Gated
All	100,00
C-	17,72
C+	82,28

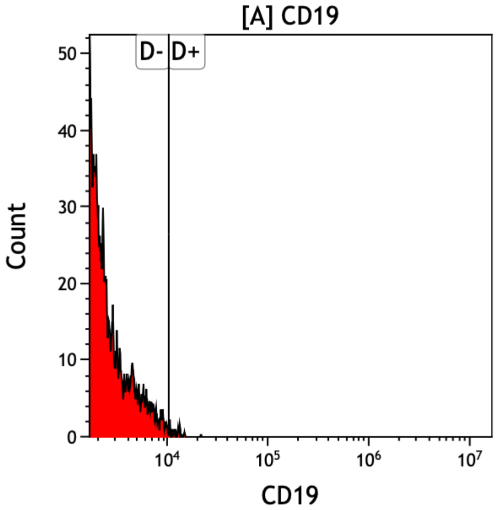

Gate	%Gated
All	100,00
D-	99,38
D+	0,62

HEALTHY DONOR MONOCYTES - 5

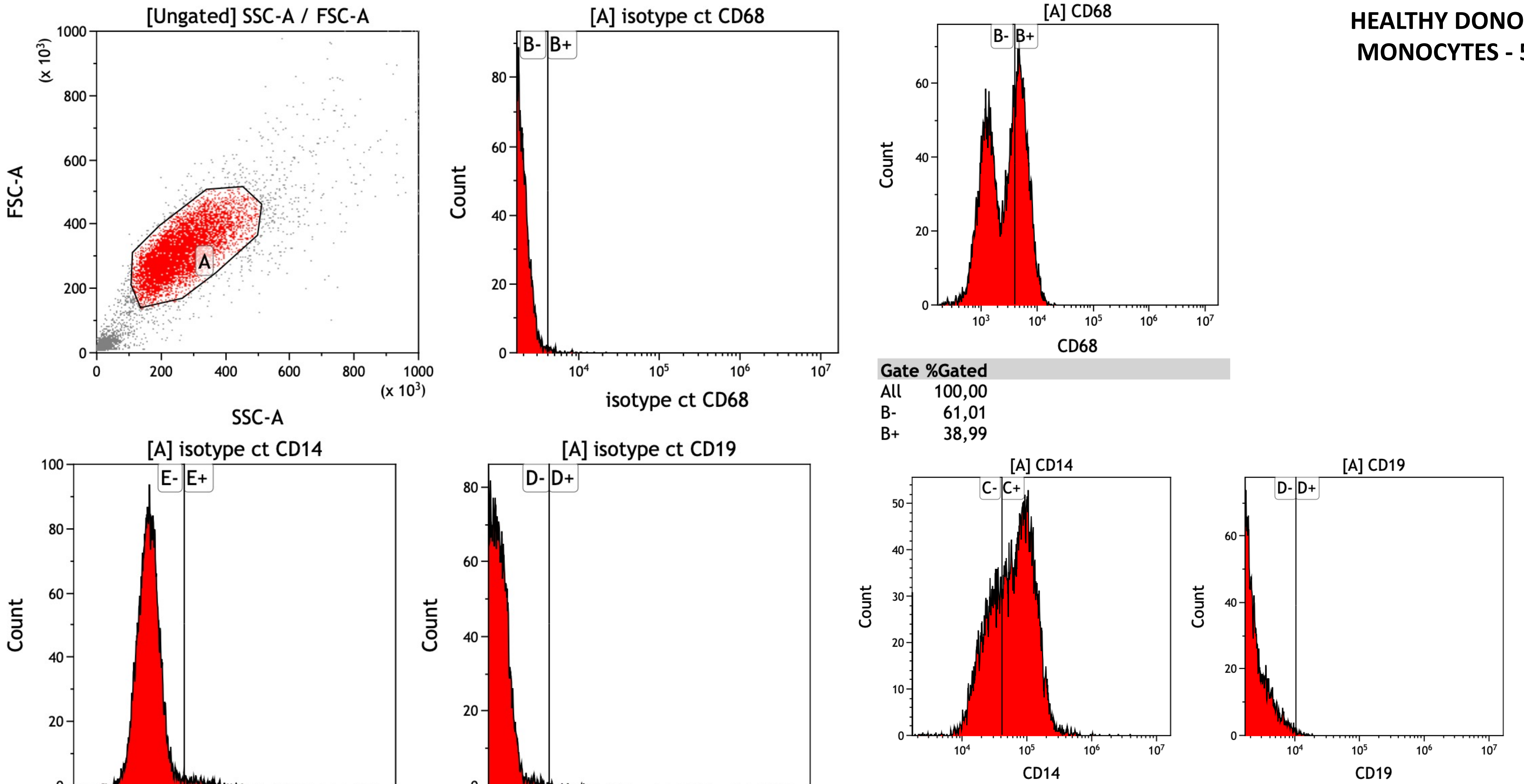

Gate	%Gated
All	100,00
B-	61,01
B+	38,99

Gate	%Gated
All	100,00
C-	33,16
C+	66,84

Gate	%Gated
All	100,00
D-	99,66
D+	0,34

HEALTHY DONOR MONOCYTES - 6

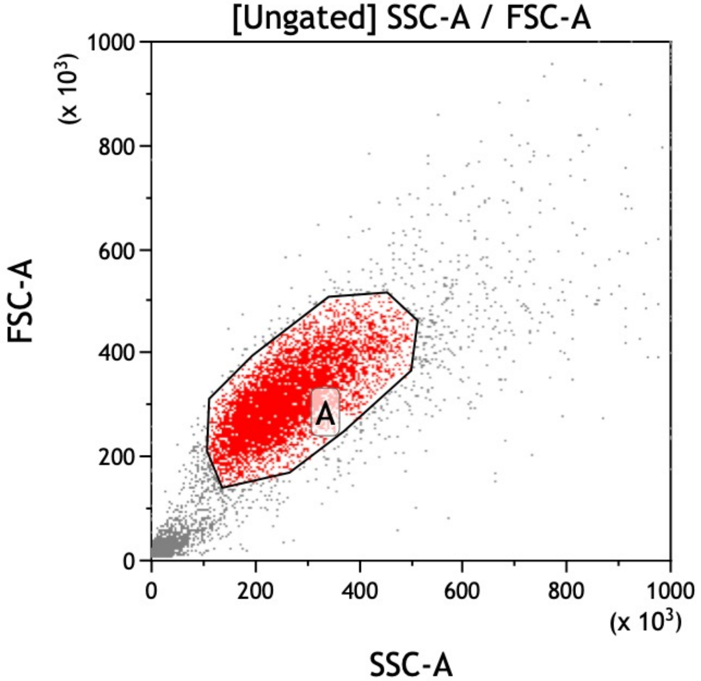

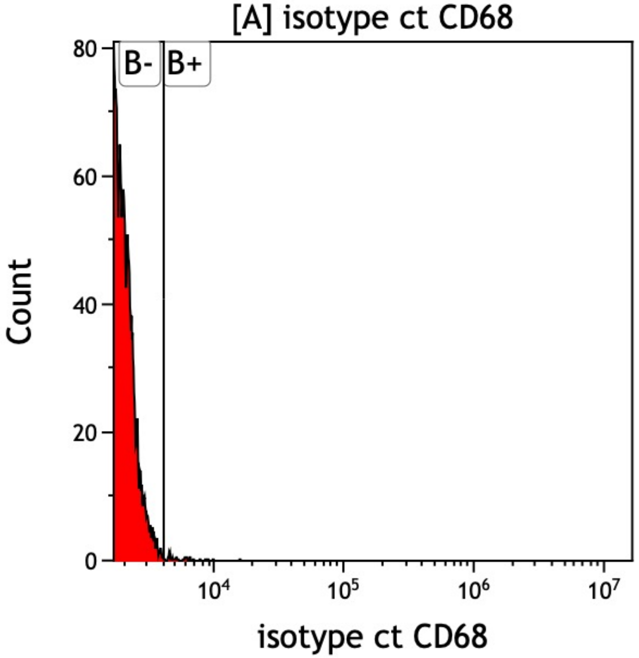

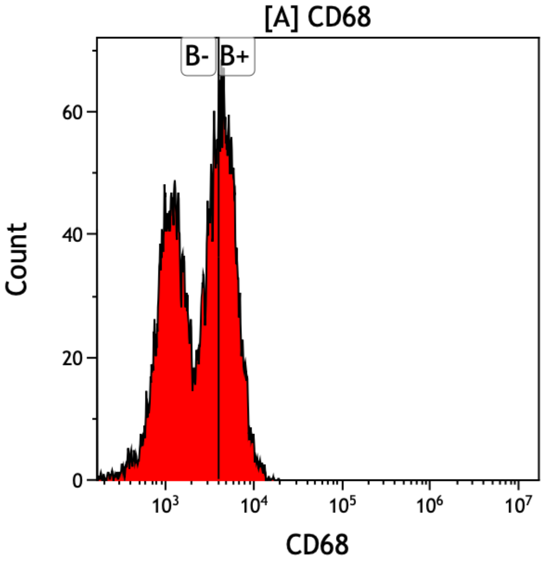

Gate	%Gated
All	100,00
B-	65,19
B+	34,81

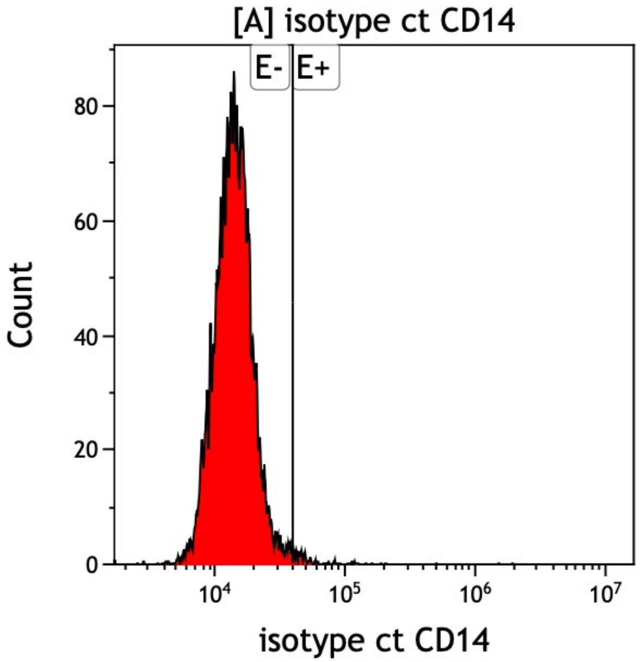

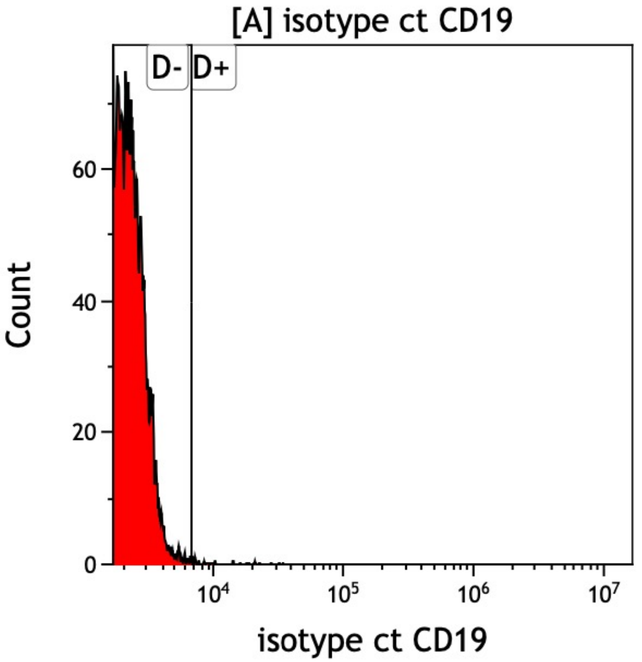

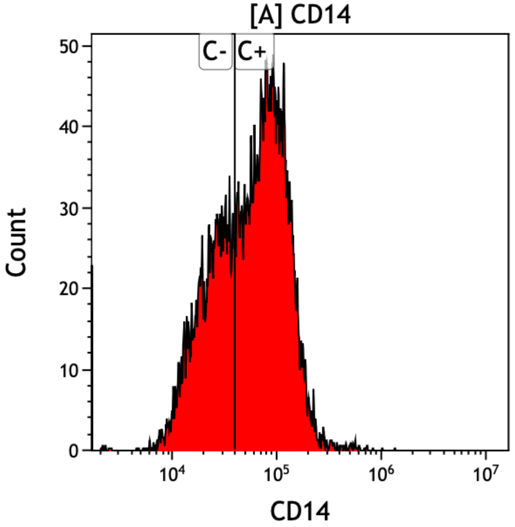

Gate	%Gated
All	100,00
C-	34,56
C+	65,44

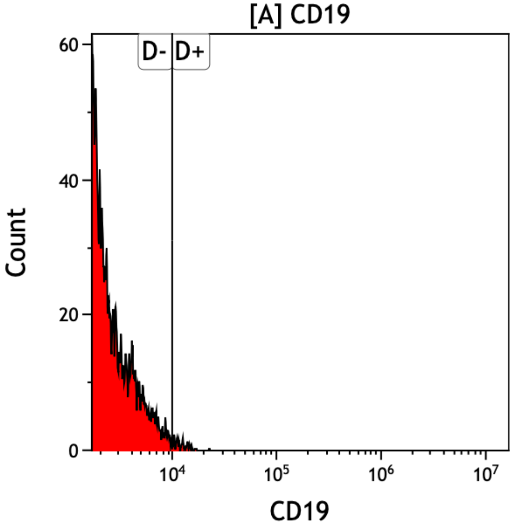

Gate	%Gated
All	100,00
D-	99,43
D+	0,57

HEALTHY DONOR MONOCYTES - 7

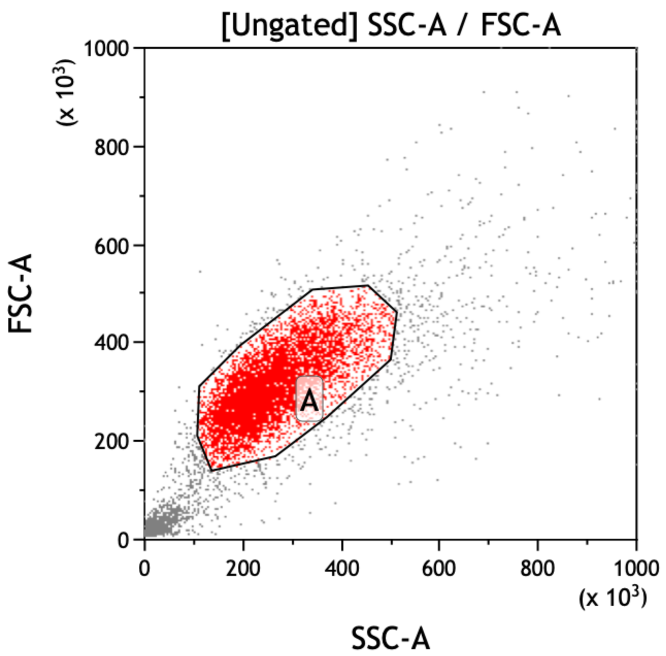

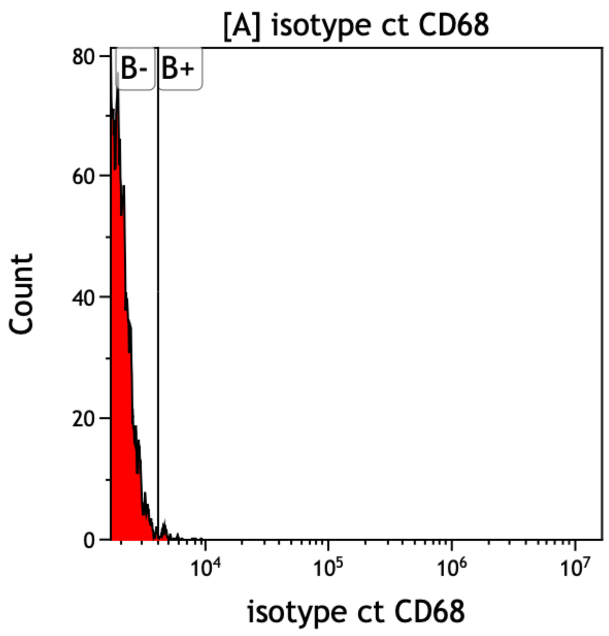

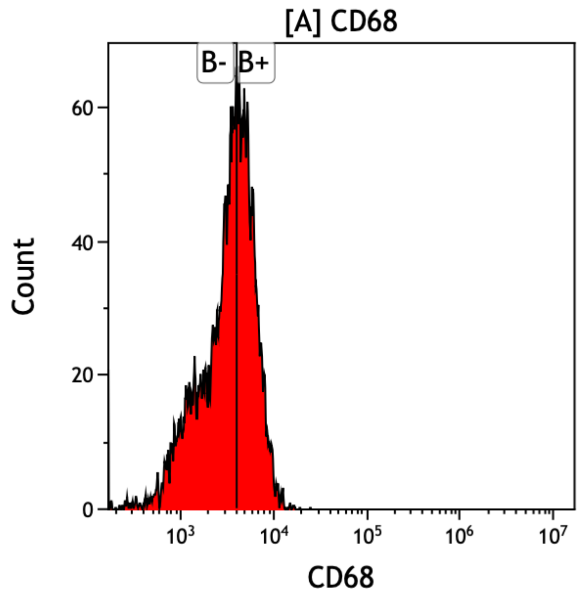

Gate	%Gated
All	100,00
B-	56,17
B+	43,83

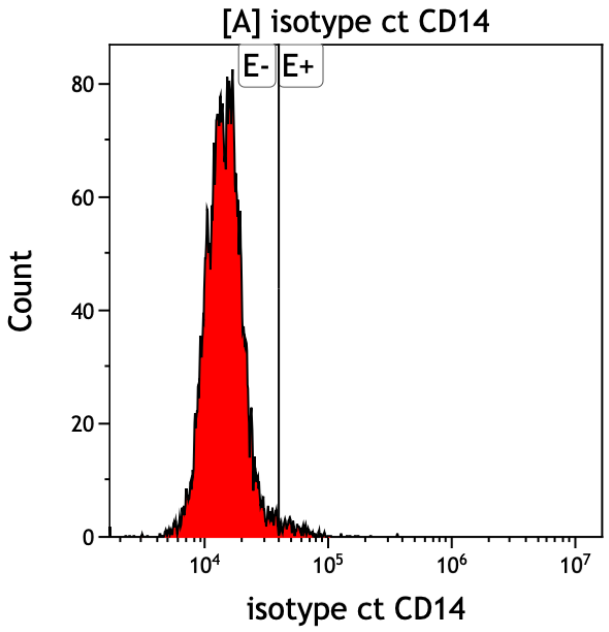

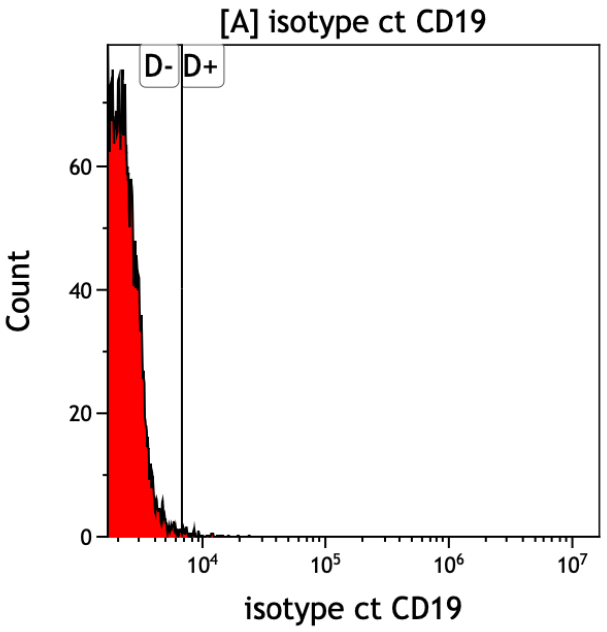

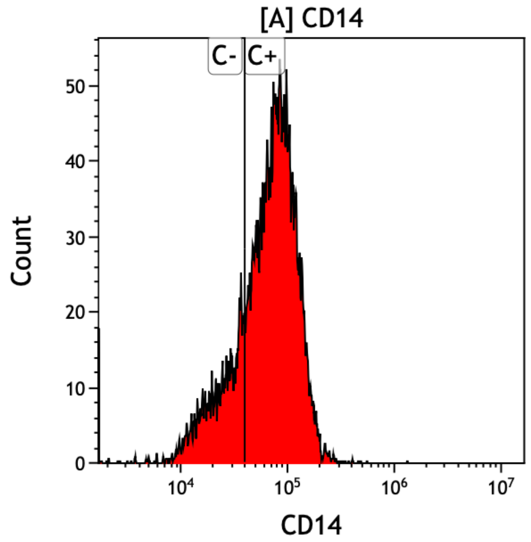

Gate	%Gated
All	100,00
C-	21,71
C+	78,29

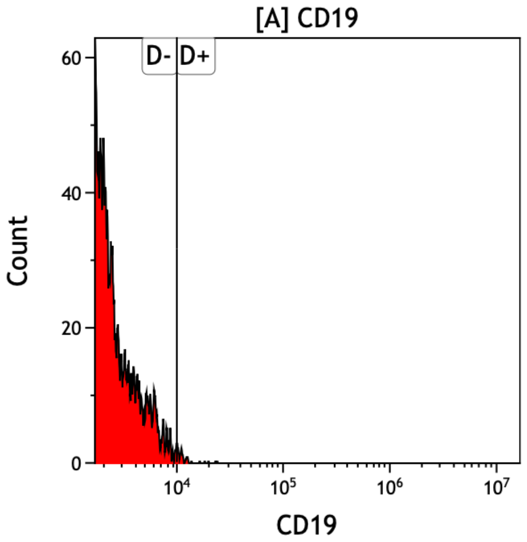

Gate	%Gated
All	100,00
D-	99,38
D+	0,62

HEALTHY DONOR MONOCYTES - 8

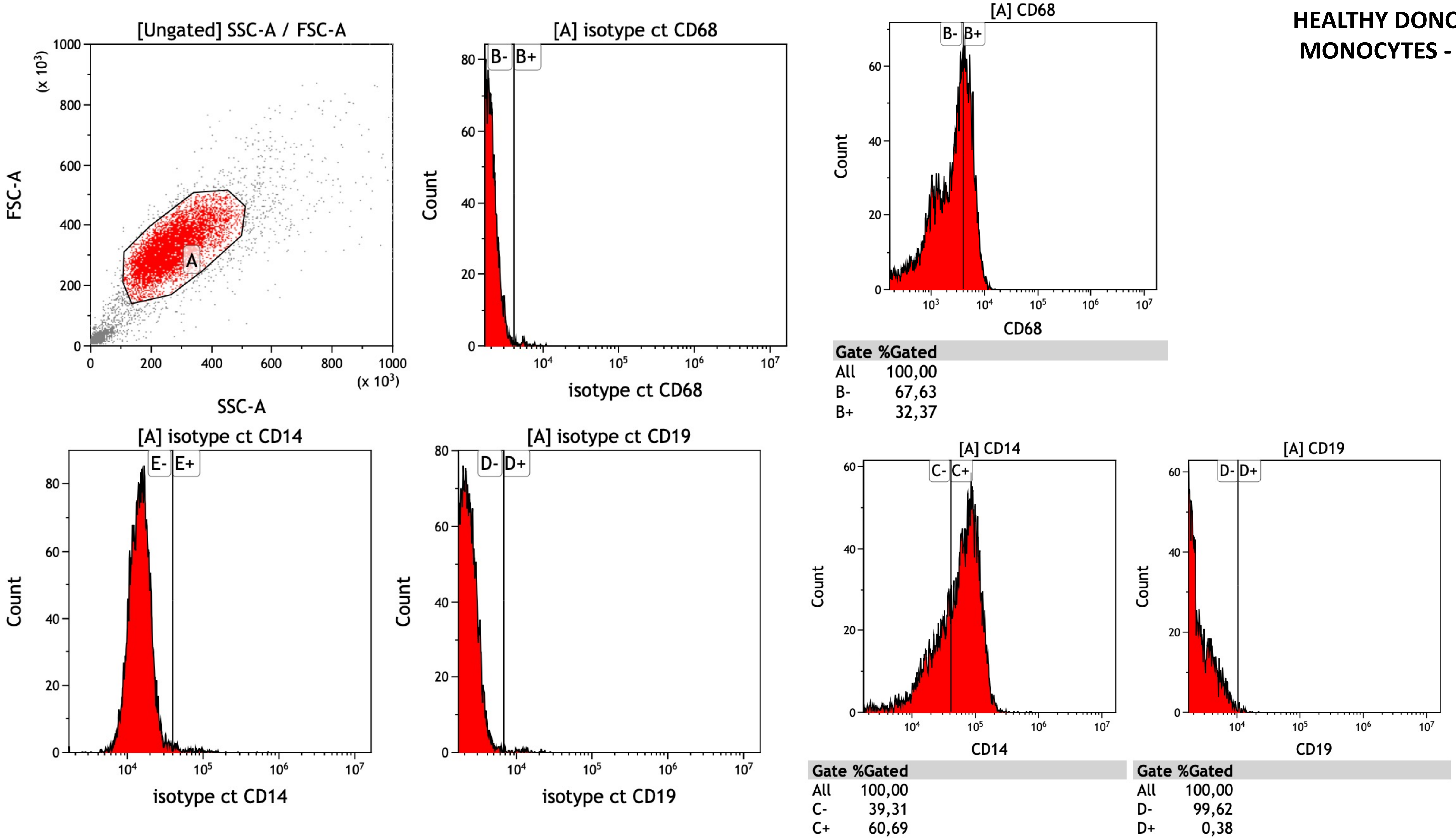

HEALTHY DONOR MONOCYTES - 9

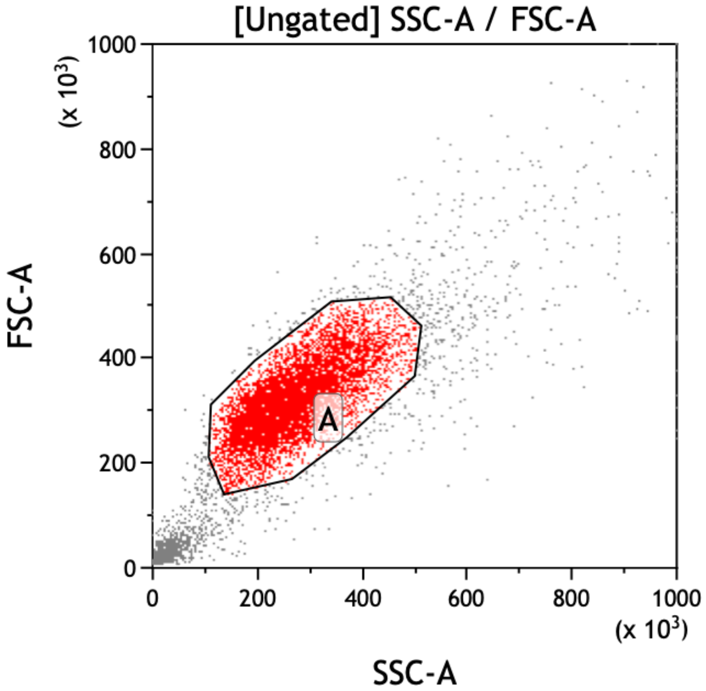

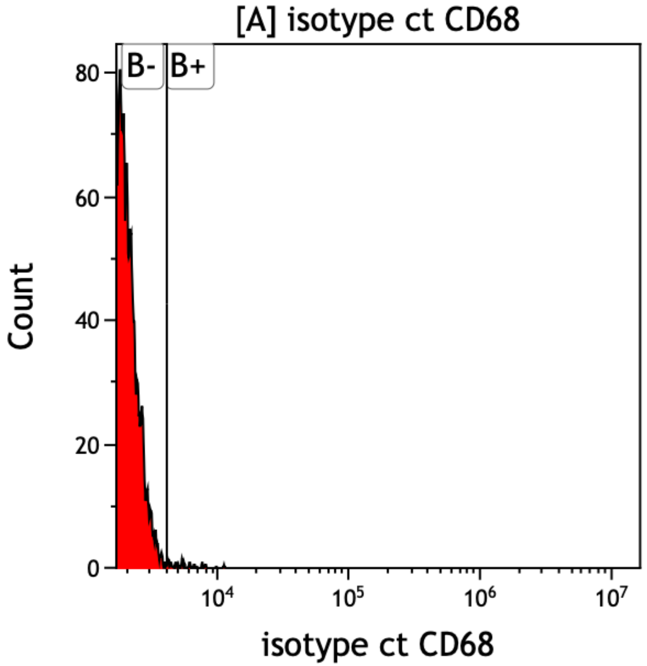

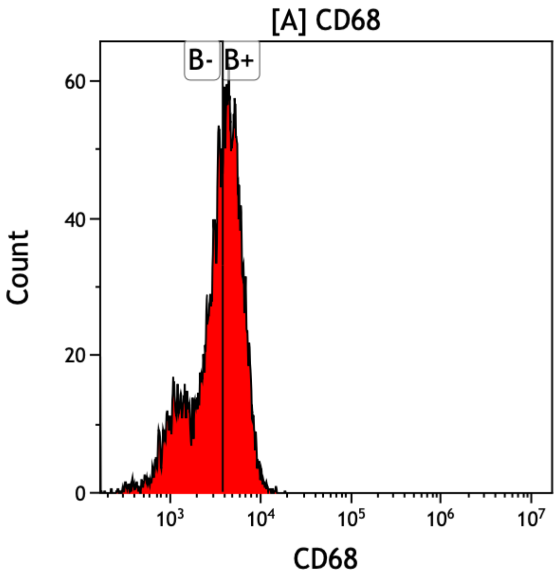

Gate	%Gated
All	100,00
B-	49,23
B+	50,77

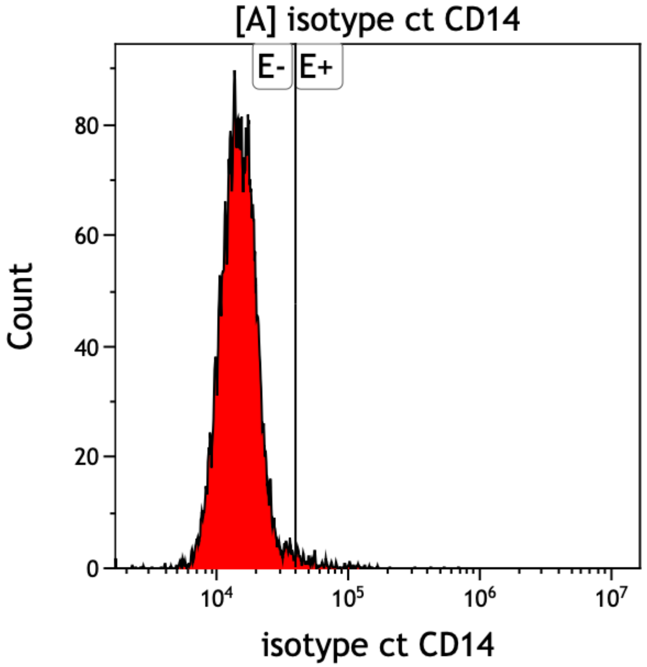

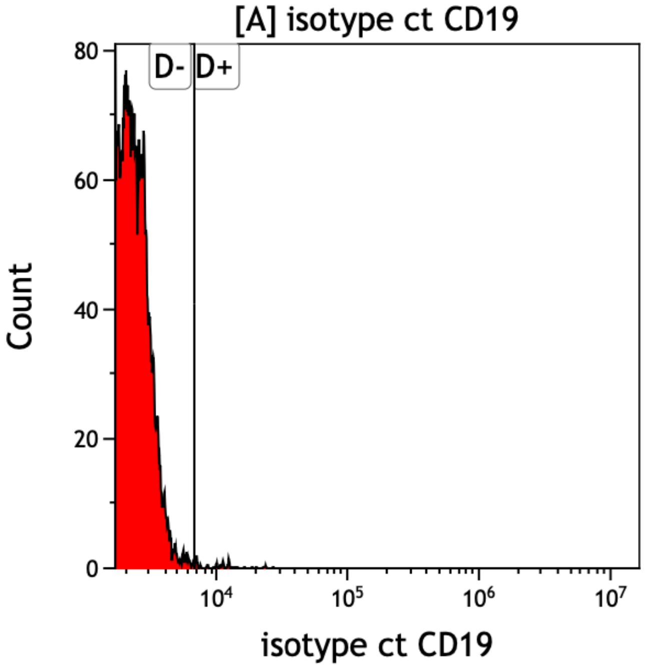

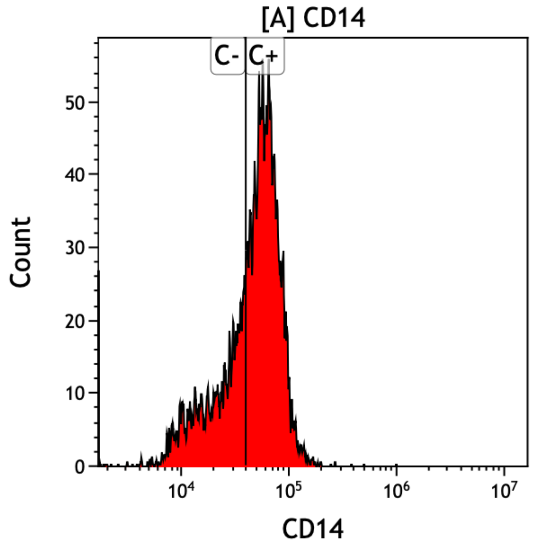

Gate	%Gated
All	100,00
C-	32,86
C+	67,14

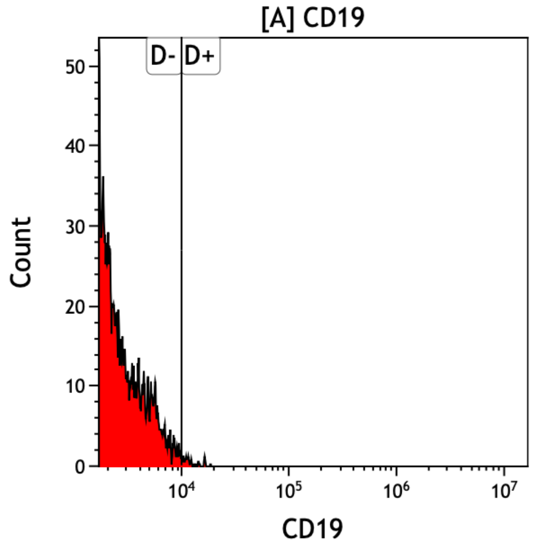

Gate	%Gated
All	100,00
D-	99,44
D+	0,56

HEALTHY DONOR MONOCYTES - 10

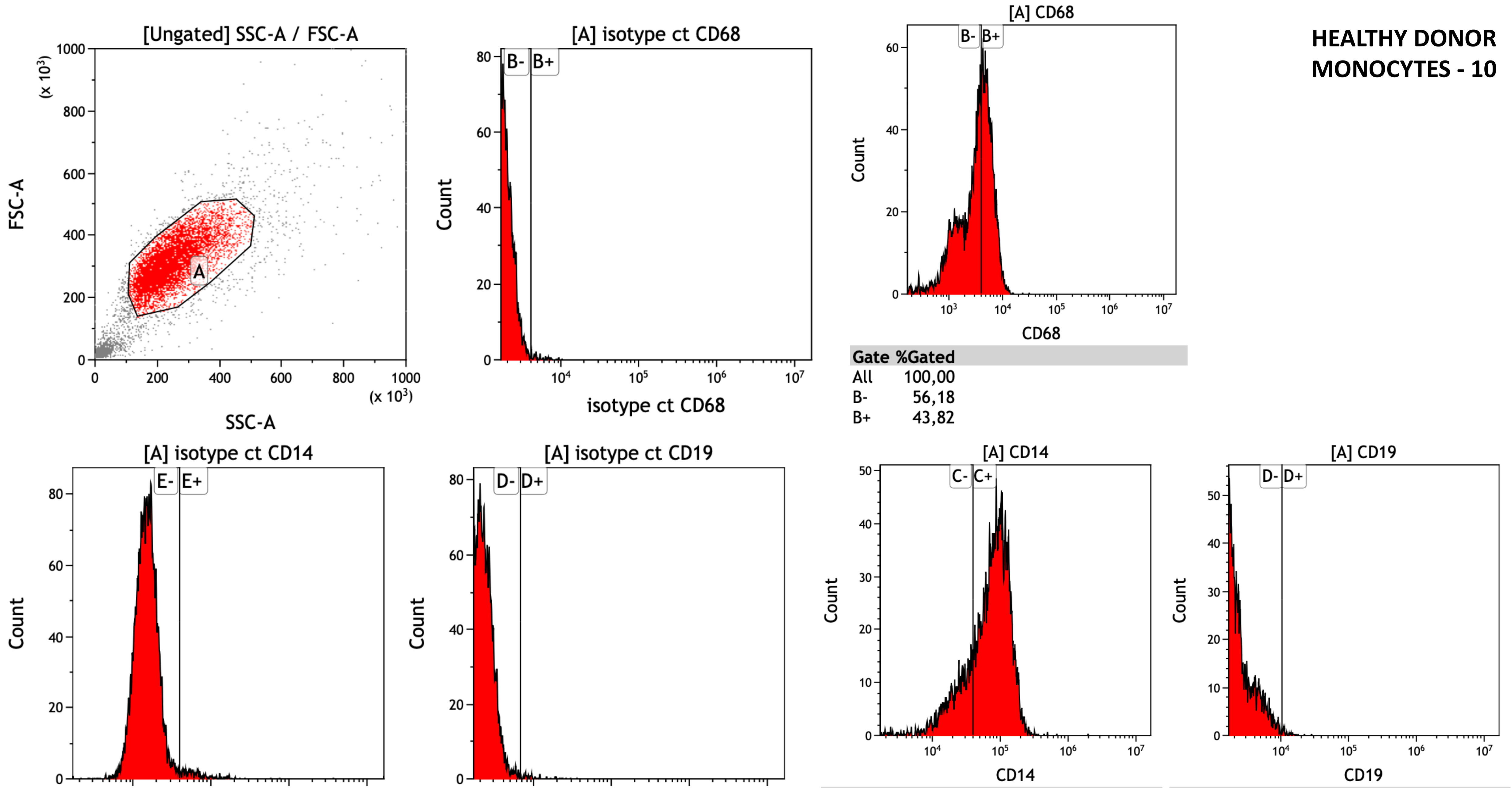

Gate	%Gated
All	100,00
B-	56,18
B+	43,82

Gate	%Gated
All	100,00
C-	24,73
C+	75,27

Gate	%Gated
All	100,00
D-	99,50
D+	0,50

Flow cytometry – CLL-B-cells + PKH67 stained-EVs

M2-THP1 EVs

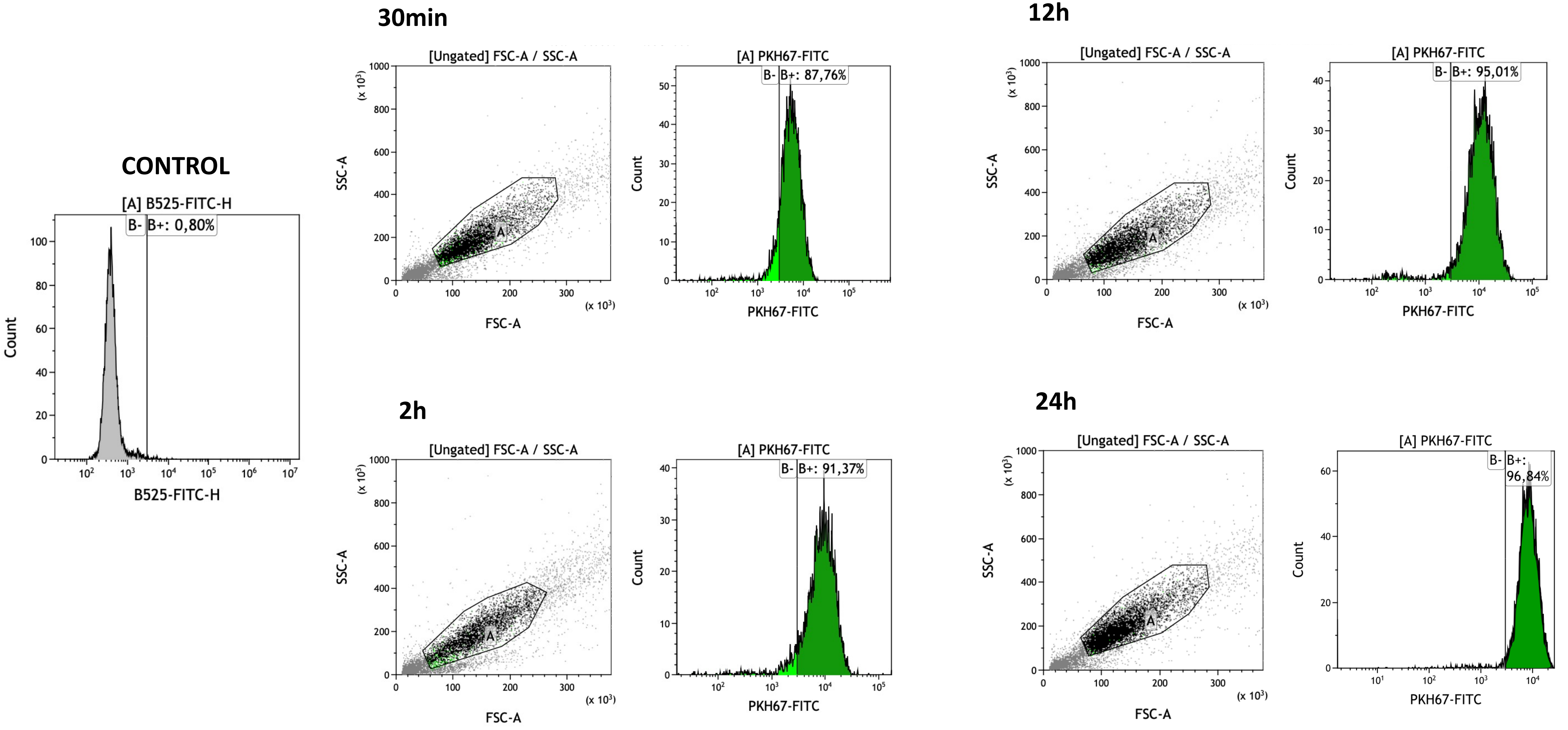

CONTROL
[A] B525-FITC-H
B-
B+: 0,80%
Count
B525-FITC-H
30min
[Ungated] FSC-A / SSC-A
SSC-A
FSC-A
(x 10³)
A
[A] PKH67-FITC
B-
B+: 87,76%
Count
PKH67-FITC
2h
[Ungated] FSC-A / SSC-A
SSC-A
FSC-A
A
[A] PKH67-FITC
B-
B+: 91,37%
Count
PKH67-FITC
12h
[Ungated] FSC-A / SSC-A
SSC-A
FSC-A
A
[A] PKH67-FITC
B-
B+: 95,01%
Count
PKH67-FITC
24h
[Ungated] FSC-A / SSC-A
SSC-A
FSC-A
A
[A] PKH67-FITC
B-
B+: 96,84%
Count
PKH67-FITC

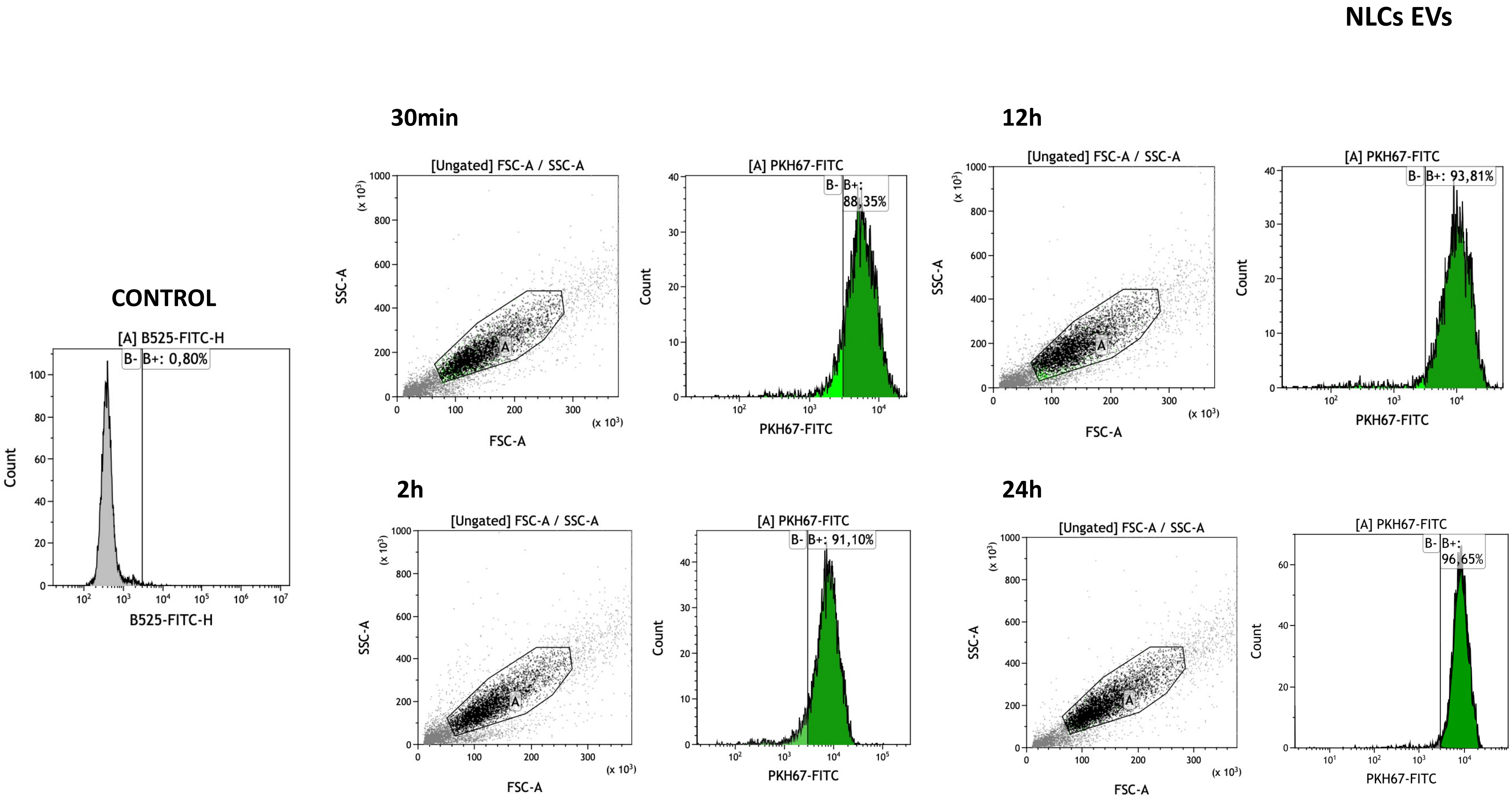
NLCs EVs
CONTROL
[A] B525-FITC-H
B-
B+: 0,80%
Count
B525-FITC-H
30min
[Ungated] FSC-A / SSC-A
SSC-A
FSC-A
A
[A] PKH67-FITC
B-
B+: 88,35%
Count
PKH67-FITC
12h
[Ungated] FSC-A / SSC-A
[A] PKH67-FITC
B-
B+: 93,81%
2h
[Ungated] FSC-A / SSC-A
[A] PKH67-FITC
B-
B+: 91,10%
24h
[Ungated] FSC-A / SSC-A
[A] PKH67-FITC
B-
B+: 96,65%

Figure 1:

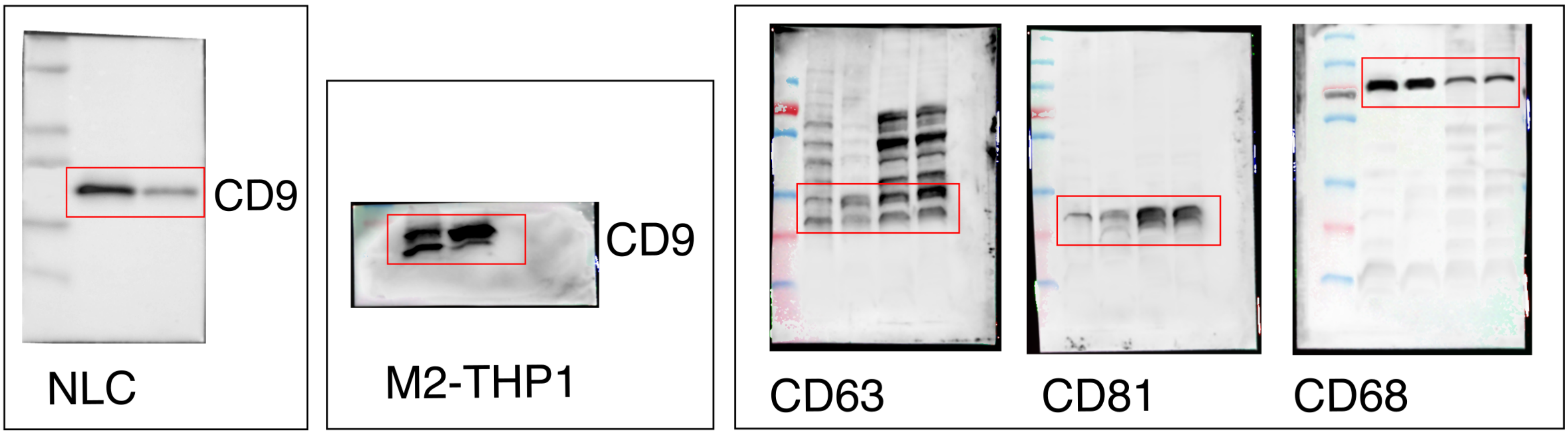

Figure 3:

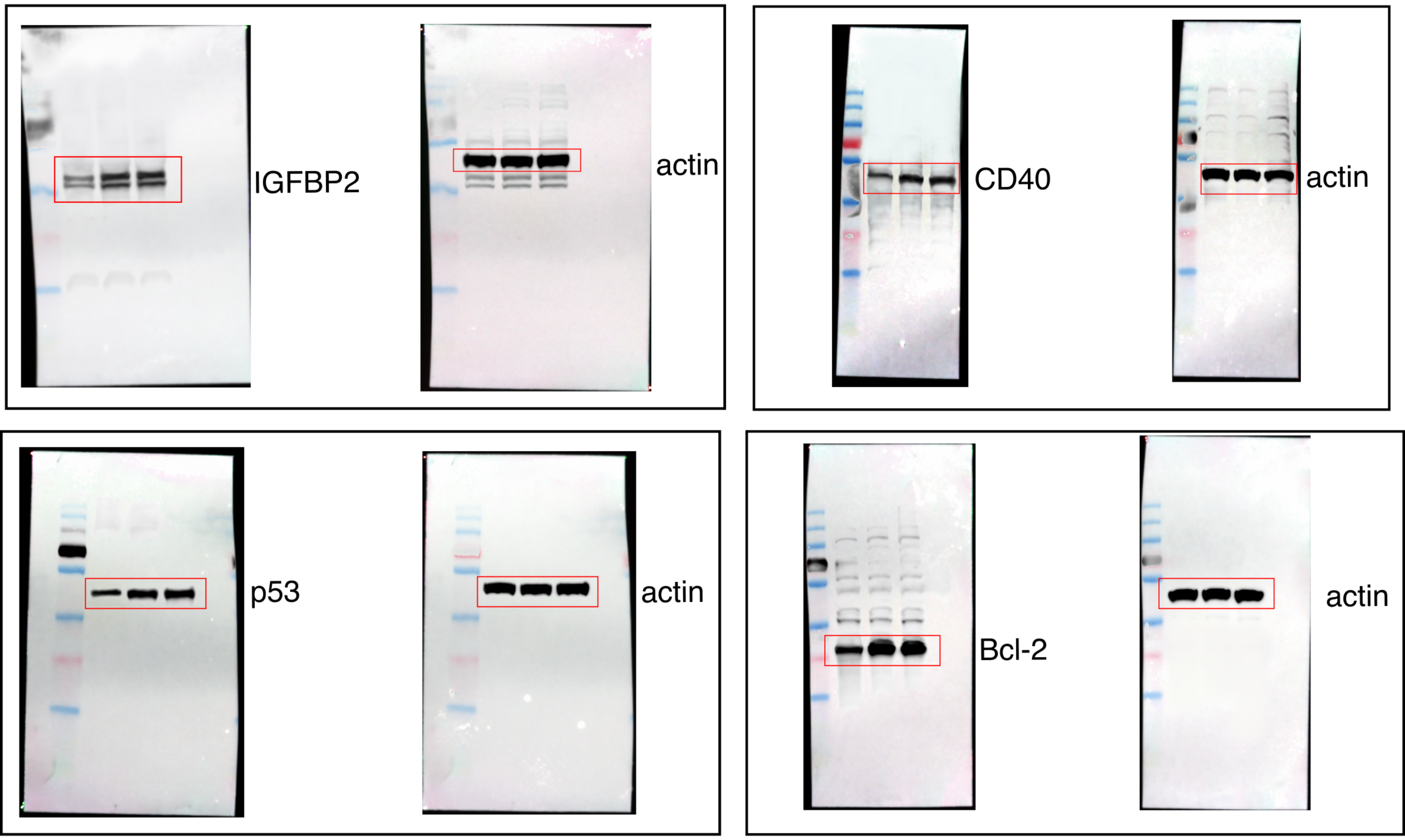

Supplementary Figure 3:

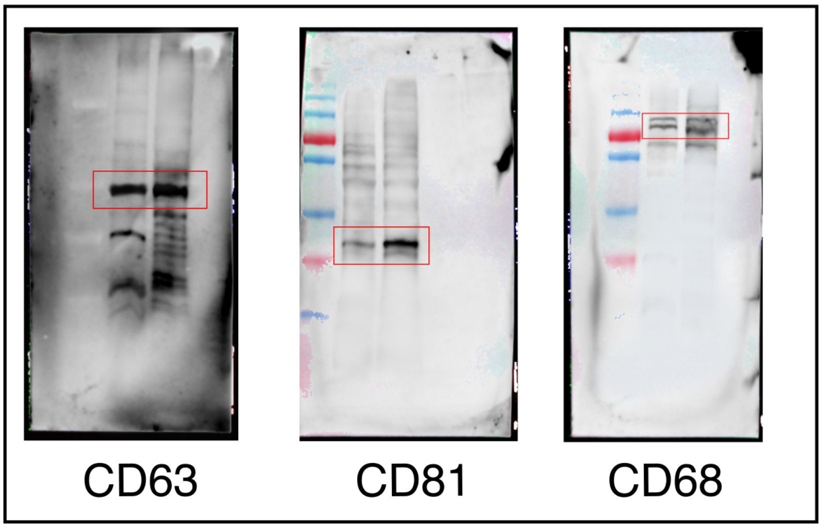

Supplement: Supplementary file 4 — Original data files [file 41417_2024_802_MOESM4_ESM.pdf]
